# Systematic Dissection of the Sequence Determinants of Gene 3’ End Mediated Expression Control

**DOI:** 10.1371/journal.pgen.1005147

**Published:** 2015-04-15

**Authors:** Ophir Shalem, Eilon Sharon, Shai Lubliner, Ifat Regev, Maya Lotan-Pompan, Zohar Yakhini, Eran Segal

**Affiliations:** 1 Department of Computer Science and Applied Mathematics, The Weizmann Institute of Science, Rehovot, Israel; 2 Department of Molecular Cell Biology, The Weizmann Institute of Science, Rehovot, Israel; 3 Department of Computer Science, Technion, Haifa, Israel; 4 Agilent Laboratories, Tel Aviv, Israel; University of California San Francisco, UNITED STATES

## Abstract

The 3’end genomic region encodes a wide range of regulatory process including mRNA stability, 3’ end processing and translation. Here, we systematically investigate the sequence determinants of 3’ end mediated expression control by measuring the effect of 13,000 designed 3’ end sequence variants on constitutive expression levels in yeast. By including a high resolution scanning mutagenesis of more than 200 native 3’ end sequences in this designed set, we found that most mutations had only a mild effect on expression, and that the vast majority (~90%) of strongly effecting mutations localized to a single positive TA-rich element, similar to a previously described 3’ end processing efficiency element, and resulted in up to ten-fold decrease in expression. Measurements of 3’ UTR lengths revealed that these mutations result in mRNAs with aberrantly long 3’UTRs, confirming the role for this element in 3’ end processing. Interestingly, we found that other sequence elements that were previously described in the literature to be part of the polyadenylation signal had a minor effect on expression. We further characterize the sequence specificities of the TA-rich element using additional synthetic 3’ end sequences and show that its activity is sensitive to single base pair mutations and strongly depends on the A/T content of the surrounding sequences. Finally, using a computational model, we show that the strength of this element in native 3’ end sequences can explain some of their measured expression variability (R = 0.41). Together, our results emphasize the importance of efficient 3’ end processing for endogenous protein levels and contribute to an improved understanding of the sequence elements involved in this process.

## Author Summary

We present a large-scale experimental investigation into sequence determinants of 3’ end mediated gene expression regulation, by measuring 13,000 designed 3’ end sequences. While 3’ end sequences contribute to expression differences through a variety of mechanisms including mRNA stability and regulation of translation, we find a predominant effect of mRNA 3’ end processing efficiency. Using extensive designed mutagenesis analysis we find that out of three functional elements described in the literature as comprising the polyadenylation signal, a single element (known as the efficiency element) is responsible for most of the effect on protein expression levels. Our work highlights the importance of 3’ end processing in expression regulation and facilitates the incorporation of the effect of this region into more complete models of DNA encoded gene expression regulation.

## Introduction

The genomic region downstream to the open reading frame (3’ end region) is commonly considered to affect expression at the post-transcriptional level through regulatory sequences that reside within the mRNA 3’ un-translated region (3’ UTR) affecting mRNA stability and translation[1–3]. In addition, mRNA 3’ end processing, a process in which the transcribing pre-mRNA molecule is cleaved and polyadenylated to create a mature mRNA molecule, is also encoded in the gene 3’ end region. The efficiency of 3’ end processing has the potential to affect gene expression in several ways. First, as 3’ end processing is coupled to transcription termination[4,5], more efficient 3’ end processing enhances the release of RNA polymerase subunits for additional rounds of transcription, thereby enhancing the rate of promoter transcription initiation[6]. Second, inefficient 3’ end processing can result in transcription read-through for a fraction of initiation events. These un-cleaved transcription events result in mRNAs with aberrantly long 3’ UTRs that are degraded in the nucleus by surveillance mechanisms reducing the number of mature mRNA molecules exported to the cytoplasm[7,8]. While all of these processes, mRNA stability, translation and 3’ end processing, were shown to potentially modulate expression, the relative contribution of each to native variability in protein levels remains unclear.

The yeast *S*. *cerevisiae* is a well-studied model organism, easily accessible for genetic manipulation and contains short intergenic regions which make it an attractive model for studies aiming to model gene expression levels using the regulatory DNA sequence alone[9–12]. Moreover, the lack of RNA interference in yeast makes it suitable for studying other basic processes that are encoded in the 3’ end sequence such as 3’ end processing. The sequence determinants of 3’ end processing have been studied by experimentally manipulating only few 3’ end sequences or by computationally looking for enriched sequences around genome-wide polyadenylation (polyA) sites (mRNA 3’ ends)[13–18]. The data accumulated from these studies resulted in a current model for yeast 3’ end processing sequence signals composing of an efficiency element (EE)- a short motif composed of TA di-nucleotides, positioning element (PE)- which is highly A rich, and the cleavage site itself which has limited sequence requirements and is usually composed of a short stretch of T[19]. These sequences are usually referred to as the polyA signal, yet to date, a systematic mutagenesis analysis aimed at directly determining the effect of each of them on protein expression and mRNA 3’ end formation is lacking. From this reason, the relative contribution of each element and the definition of its sequence specificities are in some cases vague and are often not verified by in-depth experiments. As a result, despite much research, our understanding of how DNA sequence encodes for expression typically incorporates the contribution of gene promoters alone.

To dissect the relative contribution of a specific genomic region to the overall expression level, it is not possible to use native expression levels since these represent the net effect of all regulatory layers. This challenge is usually addressed by separately fusing different regulatory sequences to a reporter gene, usually a fluorescent protein, with a constant genomic context. In addition, since each pair of native regulatory sequences differ by many parameters, using synthetic sequences that are designed to examine specific hypothesis proved to be very informative[20–29]. For example, one study dissected the effect of nucleosome disfavoring sequences on promoter expression by designing sets of promoters that differ only in the strength and number of their nucleosome disfavoring sequences[24]. Since the amount of labor and time in the generation of each individual clone and measuring its induced expression limits the scale of designed reporter gene studies, we and others[27,29] have recently developed a method to generate and accurately measure the expression induced by thousands of sequences using pooled ligations and transformations followed by reporter gene fluorescence activated cell sorting and parallel sequencing. This method was successfully utilized to study the sequence rules that govern promoter regulation in yeast[27].

Here we adopted the above high throughput approach to study the sequence determinants of 3’ end regulation. We designed a set of 13,000 synthetic 3’ end sequences *in-silico* to contain 917 native sequences, scanning mutagenesis for 217 native sequences, and various manipulations of literature curated regulatory elements. Together, our library represents the first systematic investigation into 3’ end sequence functionality at this scale. While literature describes both 3’ UTR elements that increase and decrease protein levels[30,31], we found that almost all mutations resulted in the disruption of a positive rather an inhibitory elements. Moreover, almost all native sequences contained only a single short (10–20bp) element in which mutations decrease expression by up to 10-fold (relative to 1000-fold between induced and non-induced promoter) and result in aberrantly long mRNAs. The element was rich in dTdA, similarly to the previously described EE. Our computational analysis detects that the strength of this element is partially predictive of the expression that native 3’ end sequences mediate. We further characterized the EE by showing that mutating it by a single bp or reducing the A/T content of its surrounding sequences significantly reduces expression. In addition, we show that other previously described elements have much lower effect on expression level. Our results highlight the importance of 3’ processing efficiency in maintaining protein expression levels and define the exact sequence determinants underlying this effect.

## Results

### Pooled protein expression measurements of 13,000 gene 3’ end sequences

**Fig 1:**
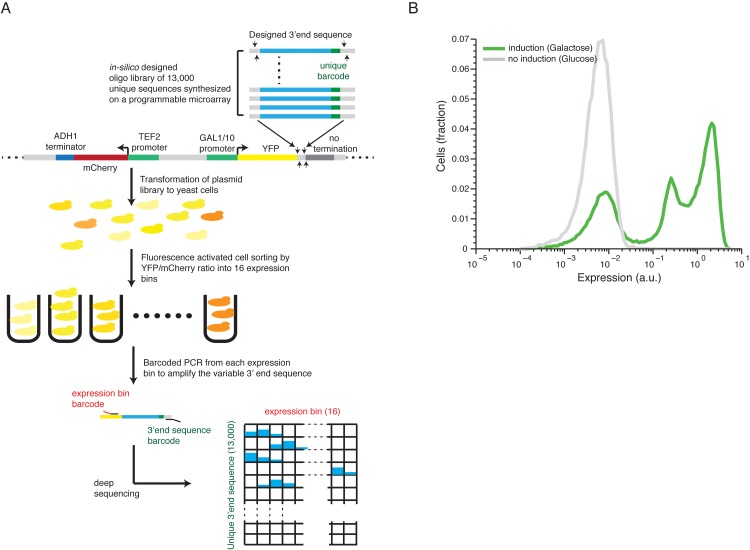
Illustration of our method and overall expression distribution. **(A)** 13,000 designed synthetic sequences were ligated into a low copy plasmid (top part). The plasmid pool was then transformed into yeast to create a heterogeneous pool of yeast cells each expressing YFP to a different level corresponding to one of the unique 13,000 cloned 3’ end sequences. The cells were then sorted using fluorescence activated sorting (FACS) into 16 expression bins by the YFP/mCherry ratio (middle). Next, the reporter 3’ end sequences of cells in each bin were amplified, using bar coded primers for each bin, and sequence barcodes was recovered using next-generation sequencing (NGS). Finally, each sequencing read was mapped to a specific 3’ end sequence and a specific bin (bottom) to achieve the distribution of cells with each synthetic 3’ end sequence across the expression bins. The distribution of each construct was fit to a gamma distribution and the mean expression value was inferred based on this fit. **(B)** The distribution of library expression values in induced and un-induced promoter states. The induced state displays a tri-modal distribution with 3 peaks corresponding to (1) non-induced promoter state (2) induced promoter state and low expressing 3’ end sequences and (3) induced promoter state with a wide range of 3’ end mediated expression.

To study the effect of a large scale library of 13,000 *in-silico* designed 3’ end sequences on protein expression, we adopted a method that we previously developed[27] (**Fig 1A**, see Methods for details). We cloned the sequences as a pool into a plasmid downstream to a yellow fluorescence protein (YFP) induced by a *GAL 1/10* promoter and upstream to a *CYC1* coding sequence followed by it’s mutated 3’UTR[32] to ensure that there will be no pre-mRNA cleavage immediately downstream the integrated sequence. After transformation to yeast cells, we measured the induced expression by fluorescence activated sorting (FACS) followed by parallel sequencing of the 3’ end sequences and inferred for each 3’ end sequence its mean induced expression level. We estimated the technical noise of our system by examining 87 groups of at least ten independent strains with identical sequences except for an 11bp barcode in their 3’ and found that the technical relative standard deviation (RSD) is 13.2% (estimated by the median RSD over all groups, **S1 Fig**). In order to evaluate the dynamic expression range achieved by switching 3’ end sequences we compared the expression distribution of our library to the expression differences of induced and un-induced Gal1/10 promoter states. We found that that the examined sequences span one order of magnitude out of three order of magnitude difference between induced to un-induced promoter (**Fig 1B**, see Methods for details). The middle peak corresponds to non-terminating 3’ end sequences (see below). We conclude that our approach can measure the effect of the 3’ end sequences on the expression level with high accuracy and over a wide range of expression levels.

### Scanning mutagenesis reveals functional elements in 3’ end sequences

**Fig 2:**
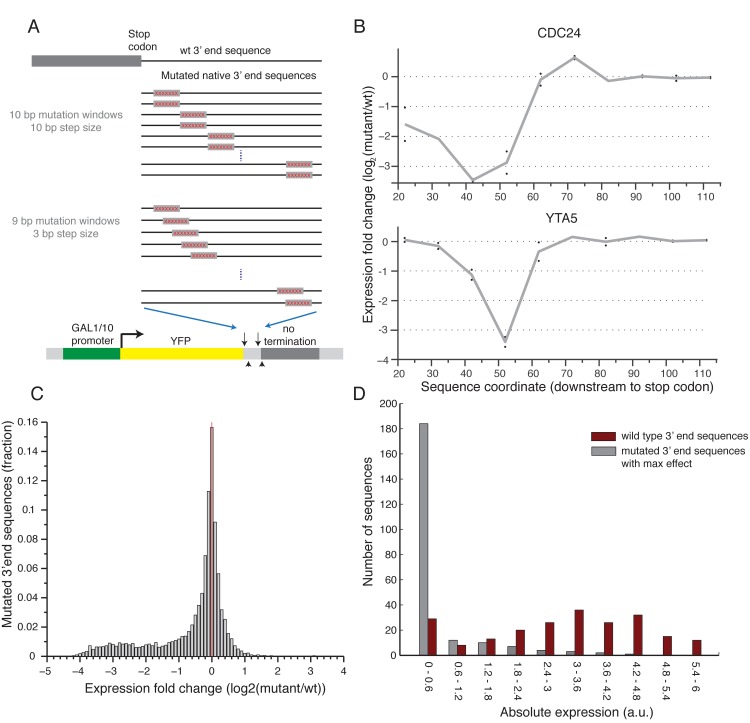
Scanning mutagenesis of native 3’ end sequences reveals critical elements required to maintain expression. **(A)** Illustration of the two scanning mutagenesis strategies used, in the upper panel two 10bp mutation windows were designed with non-overlapping 10bp steps. In the lower panel 9bp mutation windows were designed with overlapping 3bp steps. **(B)** Profile of the effect of mutations as a function of location for two genes: CDC24 and YTA5. Y-axis shows the expression log_2_ fold change compared to the wild type sequence with each point representing a single 10bp mutation window centered around the corresponding x-axis value relative to the stop codon. The gray line connects the average of each pair of mutations. **(C)** Distribution of log2 fold ratio between mutated and wild type 3’ end sequences showing a highly skewed distribution towards negative values. **(D)** Distribution of absolute expression values (a.u.) for non-mutated native 3’ end sequences (dark red) and mutated 3’ end sequences (gray). For the mutated sequences, the mutation that resulted in the largest reduction in expression was chosen for each native sequence.

To conduct a screen for functional elements in 3’ end sequences, we selected a set of 217 native 3’ end sequences for which both the main polyA site and more than 80% of the mapped sites were within the synthesized sequence[17], and designed a systematic mutagenesis set for each. We applied two systematic mutagenesis schemes: 1) Two different 10bp modifications in each position in intervals of 10bps; 2) Single 9bp modification in each position in intervals of 3bp (**Fig 2A** and Methods). In both cases we randomly mutated each base pair in the mutated window and did it twice each time ending up with a different mutated sequence to replace the original one (see Methods). Fig 2B shows an example of the expression profile of two 3’ end regions, mutated according to the first scheme. We found that the effect is primarily due to the sequence that was removed by showing that the expression induced by two 3’ end sequences with different modification at the same position is highly correlated (R^2^ = 0.77, Pearson correlation **Figs 2B and S2**). Reassuringly, we also show that mutations upstream to the measured polyadenylation site[17] have significantly stronger effect than mutations downstream (t-test p<10^–69^, 2529 upstream mutations, 1067 downstream mutations, **S3 Fig**). These results suggest that the scanning mutagenesis scheme can be used to detect functional elements in 3’ end sequences.

**Fig 3:**
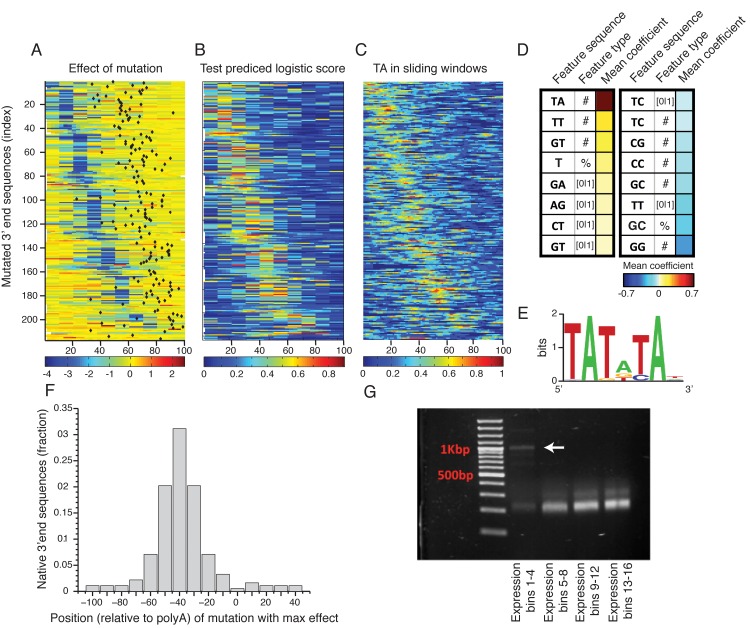
Sequence determinants of 3’ end functional elements. **(A)** Heat map showing the mean effect of a mutation as a function of location in the 3’ end sequence. Each row represents one sequence and the color represents the mean expression fold change across two replicates between the mutated and wild type sequences. Rows are sorted by the location of the maximal affecting mutation. **(B)** Heat map of predicted logistic values on a held-out test set (see main text and methods). Location of subsequences correspond to those in Fig 3A. **(C)** Frequency of AT dinucleotide, highest weighted feature in the inferred model, in sliding windows of 20bp. Location of subsequences correspond to those in Fig 3A. **(D)** Table of the features that contribute most to the classification. Color represents the mean coefficient across the 10 cross validation partitions. For each possible mono/di-nucleotide three types of features were considered: ‘[0|1]’ – a binary feature that is one if the specified mono/di-nucleotide occurs at least once in the sequence and zero otherwise, ‘#’ – a counter of the number that the specified mono/di-nucleotide occurs in the sequence. ‘%’ percent of nucleotides of the sequence that are part of an occurrence of the specified mono/di-nucleotide. **(E)** DNA sequence motif found to be enriched in the positive subsequence instances. **(F)** Distribution of distances between the location (center) of the mutation that resulted in the maximal reduction in expression and the location of the main polyadenylation site for the wild type sequence. **(G)** Results of YFP specific 3’ RACE, where each lane represents 4 expression bins. Lowest lane displays long aberrant 3’UTRs not apparent in the higher expression bins.

Next, to systematically examine the effect on expression of the different mutated sequences, we computed for each position the ratio between the mean expression of sequences that were mutated in this position and the un-mutated native sequence. Interestingly, we found almost no mutations that result in an increase in expression and many that decrease expression (22.5% / 0.76% decrease / increase expression more than two-fold, respectively), indicating that for the given growth condition almost all of the functional elements that we disturbed have a positive effect on expression (**Fig 2C**). By inspecting the profile of mutational effect across all sequences, we found that most (85%) native sequences represented in our library have a single short (10–20bp) functional element whose disruption results in a substantial reduction in expression (**Figs 3A and S4**). Moreover, mutating this element reduces expression to a narrow range of low expression values (**Fig 2D**), independent of the expression level of the non-mutated sequence (**S5 Fig**). This narrow range corresponds to a lower peak in the bimodal expression distribution observed across our whole library (**Fig 1B**). Thus, we conclude that almost all the examined 3’ end sequences contain a relatively short element that is essential for maintaining the expression level mediated by the native sequence.

Next, to identify which sequence features discriminate the functional elements in the 3’ end from the rest of the sequences we employed a machine learning approach. We divided the mutated subsequences of each 3’ end sequence into a positive and a negative set such that the positive set contained subsequences that when mutated reduces expression to the lower expression peak (expression level < 1, **Fig 2D**) and the negative set contained all other subsequences. We then learned a computational model that scores each subsequence based on its mono/di-nucleotide sequence composition features with the goal of discriminating between the sets. We used three different feature types: the number of mono/di-nucleotide, the percentage of mono/di-nucleotide and a binary indicator that is one if the mono-di-nucleotide occurs at least once. The predicted discriminative values on a test set using cross validation had good correspondence to the measured expression fold change values (**Fig 3A and 3B**), where in 90% of the native sequences, the mutation with the strongest effect was correctly classified as a positive element (on holdout test data, see Methods). We examined the contribution of sequence features to the model predictions (**Fig 3D**), and found that TA dinucleotide stands out as the main contributor with a higher mean coefficient value than all other features. In addition, TA dinucleotide frequency corresponds to the positive elements (**Fig 3C**) and in line with this, a discriminative motif finder[33] identified a TA rich motif (**Fig 3E**) enriched in the positive elements that is highly similar to the previously characterized efficiency element (EE)[13].

To validate these results and show that the reduction in expression level is also at the level of mRNA expression we chose 8 3’ end sequences from the library (**S4 Table**) and cloned them individually together with their mutated sequences (chosen to be the mutated sequence with the maximal reduction in expression level). We then measured YFP level (YFP normalized by RFP over the exponential growth phase as in Zeevi et al.[34]) and YFP mRNA levels to show validation of the library in an arrayed format and also show that reduction in expression can also be observed at the mRNA level using qPCR measurements (**S6A, S7A and S7B Figs**). As our selection of 3’ end sequences was limited by synthesis length, it is possible that longer 3’ UTR would not display a similar result; we thus chose a set of 9 additional 3’ end sequences of longer length (**S4 Table**) and cloned them individually into our library backbone expression plasmid. These sequences were chosen to have well defined polyadenylation sites. In addition we also computationally identified the TA element (using the model learned on the library data) and cloned mutated versions of these 3’ end sequences. Reassuringly, we observed a similar result for 5 out of the 9 sequences for both YFP measurements (**S6B Fig**) and mRNA levels (**S7C and S7D Fig**). The 4 additional sequences showed a weaker reduction in expression levels due to the mutation, yet all of these had lower expression levels of the wt sequences indicating that either we did not clone the full regulatory sequence or these sequences has different polyadenylation signals than the ones we have predicted. Together, these results show that, at least for the subset of native 3' end sequences examined in this study, the efficiency element is the main sequence element important to maintaining 3’ end mediated expression levels, and that these elements can appear either in a specific 6mer motif (**Fig 3F**) or as a more tolerant 10–20bp TA rich region.

### Reduction of expression due to EE deletion is a result of pre-mRNA miss-cleavage

To gain insight into the mechanism responsible for the reduction in expression due to EE mutagenesis we studied the location of the polyadenylation site (mRNA end) relative to the mutational profile that we found using scanning mutagenesis. We first mapped polyadenylation sites to our wild type (non-mutated) sequences using published data[17]. We found that the location of our maximal effecting mutation tends to peak about 40bp upstream of the main polyadenylation site (**Fig 3F)** similar to the EE that was previously characterized on a small set of genes[13,19]. To measure the length of the 3’ un-translated region (3’ UTR) of our mutated sequences we performed 3’ rapid amplification of cDNA ends (3’ RACE) using a primer specific to the YFP and polyA tail (see Methods). This procedure amplified only 3’ UTR sequences of the library mRNA molecules. We first analyzed the pooled lengths of our library following sorting into four expression bins. The low expressing bins, corresponding to the distinct low peak in our whole library expression distribution, displays aberrantly long 3’ UTRs, suggesting that at least part of the mechanism that underlies the reduction in expression is that mutating the dTdA elements drives the polyA signal non-functional resulting in miss-cleavage and a long 3’UTR (**Fig 3G**). In contrast, it seems that other elements described in the literature[19,35,36] have a much lower effect on 3’ end mediated expression (**S8 Fig**).

### Variation in efficiency element sequences partly explains variation in protein levels between native 3’ end sequences

**Fig 4:**
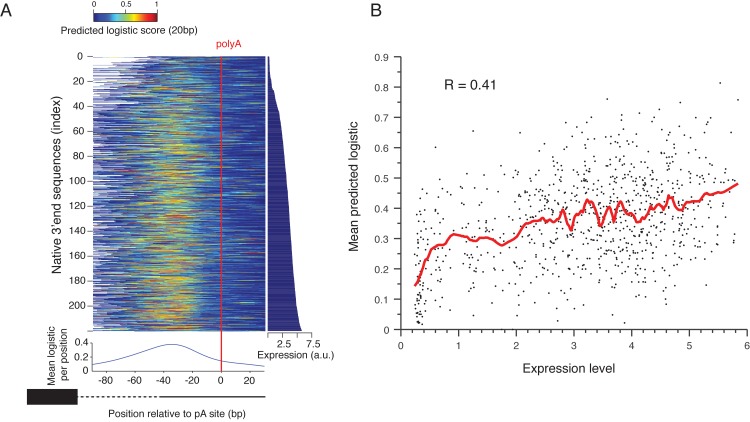
Prediction of polyadenylation signals in native sequences. **(A)** Native sequences are aligned by the main polyadenylation site and ordered by the expression values (right panel). The color indicates the predicted logistic values using the classifier learned on the scanning mutagenesis set. The lower panel shows the mean predicted logistic in a 20bp sliding window (centered) relative to the polyadenylation site. **(B)** Mean predicted logistic in a 20 bp window, centered around the peak from Fig 4A on the y-axis versus expression levels in the x-axis. The red line shows a smoothing line with 50 instances window.

To study the dynamic rage and sequence determinants of expression differences mediated by native 3’ end sequences, we designed into our library a set of 917 native 3’ end sequences chosen to contain both most (>80%) and the main polyadenylation sites within the first 102bp[17], the endogenous genes associated with these sequences spanned a wide range of expression values[37](see Methods). We found that these sequences span a dynamic range of 20 fold in expression values (**Fig 4A**, right panel). In comparison, a recent work showed that native yeast promoters have expression levels that span over 3 orders of magnitude[38]. This suggests that although promoters are the major regulators of gene expression, 3’ end sequences may play an important role in their fine-tuning. In support of this view, we found that our measured 3’ end sequences effect on expression has a low yet significant correlation with mRNA abundance^37^, protein abundance[39] and mRNA half life[40] (**S9 Fig**, R = 0.23, 0.2, 0.25 P<10^–12^, 10^–8^, 10^–11^, Pearson correlation).

To test the ability of the sequence determinants of 3’ end sequence that we learned to explain the induced expression we examined the ability of our classification model that was learned from the scanning mutagenesis set, to explain the effect of native 3’ end sequences on expression. We applied it to consecutive 10bp subsequences of each native sequence (**Fig 4A**). We found that the predicted values peak about 38bp upstream of the measured polyadenylation site[17] (**Fig 4A**, bottom panel) and that the mean predicted value of 20bp centered around this peak is highly correlated with expression level of these sequences (R = 0.41 P<10^–30^, Pearson correlation **Fig 4B**). This suggests that variation in EE strength, as captured by our model, is partially responsible for the observed differences in expression of native promoters, probably due to differential efficiency in mRNA 3’ end processing.

### Efficiency element activity is highly sensitive to single bp mutations and correlates with surrounding A/T content

**Fig 5:**
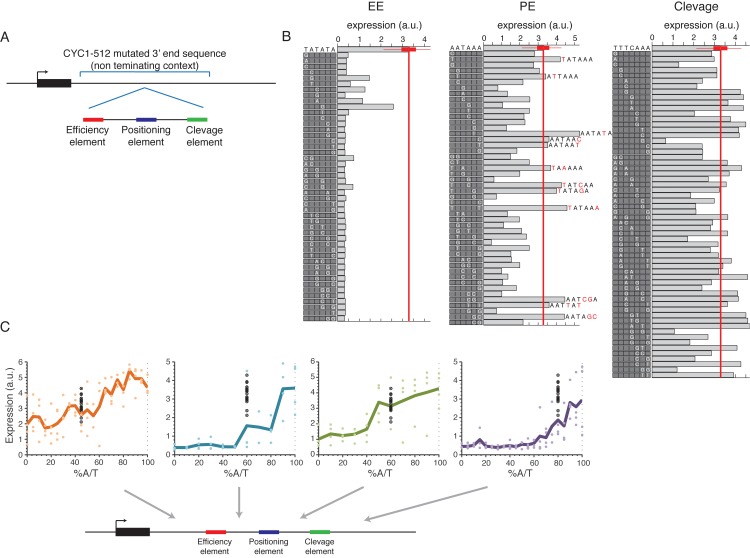
Systematic mutagenesis of a designed synthetic terminator. **(A)** Illustration of the construct design: a minimal terminator sequence was embedded within a mutated non-terminating 3’ end sequence from the CYC1-512 3’ end region. **(B)** All possible single bp mutations in the three elements EE, PE and cleavage on the left, middle and right panels, respectively. Boxes on the left of each panel show the mutated sequences with a highlighted white letter representing the location and exact mutation relative to the wild type sequence shown on the top. Bars show the expression value of each sequence. **(C)** Expression as a function of context A/T content. Each point represents a mutated sequence with A/T content of the relevant sequence region on the x-axis and expression on the y-axis. Black points show the expression of the non-mutated sequence with different barcodes. Mutated regions are: (1) upstream to EE (2) between EE to PE (3) between PE to cleavage and (4) downstream to cleavage, corresponding to the panels from left to right.

To study the sensitivity of EE, and other sequence motifs (PE, cleavage) described in the literature[19] to point mutations and to the local genomic context, we designed 3’ end sequences in which the consensus of the functional elements were embedded within the CYC1-513 3’ end mutant sequence (**Fig 5A**). This mutated 3’ end region has a 38bp deletion that was shown to result in no pre-mRNA cleavage and polyadenylation[41]. We replaced the deleted 38bp with a minimal synthetic terminator that was shown to be sufficient to promote cleavage and polyadenylation[42], and contains EE, PE and cleavage site consensus sequences embedded within a randomly chosen sequence. We tested the sensitivity of each of the three elements to single/double bp mutations using a set of sequences in which in each sequence a single/double bp mutation was introduced to one/two of the positions of one of the elements, together covering all possible mutations (**Fig 5B**). We found that the EE in this specific form and context is highly sensitive to single base pair mutations such that 89% of the mutations decreased expression to the lower peak of expression values observed in Fig 1B (**Fig 5B, left panel**). PE shows lower sensitivity to mutations, yet mutations still resulted in reductions in expression (**Fig 5B,** middle panel, P<10^–2^, **S10 Fig**). Notably, all mutations that add TA dinucleotides to PE increase expression (**Fig 5B**, middle panel, 6 of the 12 mutations that increase expression increase TA, P < 3*10^–3^). This increase is relatively small yet the non-mutated sequence is already very highly expressed. Mutations in the sequence that was defined as the cleavage site did not have any significant effect on expression (**Figs 5B and S10**). In an attempt to generalize these results to native sequences, we preformed similar mutagenesis scheme in four native 3’ ends in which we identify the three elements computationally and found similar results in some of the cases (**S11 and S12 Figs**). The cases that did not show a similar effect might be explained by alternative functional elements in its sequence.

Next, we tested how the efficiency of EE and the other elements are affected by the sequence context using designed sequences in which the sequences that flank each element were randomly mutated to achieve different A/T content levels (see Methods). We found a high correspondence between high A/T content and high expression levels in all tested regions in the 3’ end sequence (**Fig 5C**) in accordance with previous results that we obtained on a smaller set of native sequences[43]. These results add to our small scale results by establishing a causal relationship between increased A/T content and increased expression and, since the sequences were randomly mutated, show that it is indeed the A/T content itself and not specific A/T rich elements in these regions that is important. Moreover, we performed the same type of analysis on the four native sequences in which we computationally identified the three different elements and randomly mutated the sequences between them as described above. We found similar results in most regions (**S13 Fig**). We conclude that a complete EE is required for mRNA 3’ end processing but that its efficiency depends, among other things, on the A/T content of the surrounding sequences.

## Discussion

We presented here a large-scale systematic experimental investigation of the effect on expression of regulatory elements in gene 3’ end sequences, by measuring the expression driven by 13,000 fully designed 3’ end sequences. By manipulating native sequences we were able to show that the dominant effect of yeast 3’ end sequences on expression is through a positive 10–20bp regulatory element, usually one per gene, that is rich in TA dinucleotides and is required for pre-mRNA processing. We identify this element as similar to the EE, previously described using only a few genes, and show that it is highly sensitive to mutations and the surrounding sequences' A/T content. We show that while literature[44] describes a more complex sequence polyA signal comprising of a few sequence elements, elements other than the EE have a minor effect on protein expression (**S8 Fig**). Our analysis suggests that the TA content of this element determines the efficiency of 3’ end processing and as a result fine-tune gene expression levels over a 20-fold range. These results join recently published papers[6,7] in highlighting the importance of 3’ end processing efficiency in the determination of protein expression levels.

Given that 3’ end region is expected to affect expression levels in multiple positive or negative ways ranging from mRNA 3’ end processing, mRNA stability to translation rates, we expected to find a mixture of several regulatory elements that effect expression level. It is therefore surprising that the expression level of our sequences was highly dominated by a single positive sequence element that we showed to be related to mRNA 3’ end processing. In accordance with this element’s role in pre-mRNA processing, the binding motif of Nrd1[45] which is known to be associated with end processing of snoRNA and srRNA significantly peaks at the position of the EE element (p < 10^–4^, **S14 Fig**). Moreover, we also used our ability to completely design the tested sequences in order to test the effect of other known regulatory mechanisms. We measured the expression mediated by 68 known RNA binding proteins (RBP) motifs (identified computationally[46]) in open versus close 2D RNA structure contexts (**S15 Fig**). However, in contrast to our expectation, the accessibility of the RBP element did not significant effect expression and the differences between elements could be mostly explained, similar to EE, by their TA content (R = 0.71, **S15C Fig**).

The main limitation of our method is the limited length of ~100bp of our designed 3’ end sequences, a limitation imposed by the DNA synthesis method that we employed. This limited our selection of native 3’ end sequences to maximal 3’ UTR length of 102bp (38% of yeast genes). Our results may thus be biased towards short 3’ UTR genes and it is not clear what is the predictive power of the identified TA rich elements that we found in longer 3’ end sequences. It will be interesting to test whether genes with longer 3’ UTRs that were shown to encode several alternative polyadenylation sites[18] also encode alternative types of 3’ end processing elements. Another limitation of our method is our inability to differentiate between the different regulatory layers, since our experiment measures the protein levels. The question of dissecting the exact regulatory layer (in contrast to the exact regulatory sequence) becomes even more complicated with recent observations that the different layers of regulation are interconnected and affect each other[47–52]. Even though we demonstrated that the EE reduces expression by creating long mRNAs, we cannot establish whether this reduces the mRNA stability, affects transcription initiation rates, or both.

In summary, our results demonstrate that pre-mRNA 3’ end processing is not only a crucial layer in the cascade of events leading to mRNA maturation but also has the potential to act as a regulatory mechanism by which yeast 3’ ends affect expression. We demonstrated that although this effect is secondary to the promoter in scale, it can nevertheless change expression up to 20-fold. In addition, we presented an experimental approach that can be used for additional studies of 3’ end functionality and open the route for more accurate modeling of the effect of this genomic region on gene expression.

## Materials and Methods

### Strains library construction and measurements

The 3’ end sequences library was constructed and measured as described in Sharon et al.[27], except for the following changes. The sequences were designed *in-silico* and synthesized as 150bp ssDNA oligos by Agient[53]. Each 150bp oligo was designed to contain a 102bp variable region flanked by shared short (18/19bp) sequences that enabled library amplification and cloning using two unique sites for restriction enzymes: SexAI in the 5’ and AvrII in the 3’ (**Fig 1A**). The low copy plasmid to which the sequences were integrated contained two fluorescence proteins, mCherry driven by TEF2 promoter and terminated with ADH1 terminator, and a YFP gene driven by an inducible GAL 1/10 promoter (**S16 Fig**). The integration site was designed to reside downstream to the YFP coding sequence followed by part of the CYC1 coding sequence and mutated 3’ untranslated region (UTR) to ensure that there will be no 3’ end processing signals immediately downstream to where the library was integrated.

Following library 16 cycles of PCR amplification (Herculase II Fusion DNA Polymerases, Agilent) the library was gel extracted, digested with restriction enzymes, ligated to the plasmid and transformed into electrocompetent Escherichia coli (Lucigen E.cloni 10G) by electroporation to create a plasmid library with high efficiency cloning. We plated 12 transformations on four 9cm plates each and harvest 200,000 colonies per plate. Harvest bacteria were pooled together and maxi prepped (QIAGEN) to get a plasmid library. To create the yeast library we did 22 transformations of 0.5ug of plasmid DNA into 10^8 yeast cells (OD ~1.3) (Y8205 strain) and pooled them together. The cells were then grown to stationary phase and regrown to mid-exponential phase in 20% Galactose medium, gated to contain one plasmid copy (as in Sharon et al.[27]) and sorted into 16 expression bins according to their YFP/mCherry ratio. Following sorting, cells were re-grown to stationary phase and 5M cells from each bin where sampled for multiplexed colony PCR and parallel sequencing using Illumina Hi-Seq 2000 sequencing.

### 3’ end sequences library design

The library was designed using similar methodology as Sharon et al.[27] Each sequence was composed from a background sequence (also referred as “context”, **S1 Table**) in which sequence element (**S2 Table**) were inserted in a specific position (on a 3’ to 5’ scale), usually by replacement of the context sequence. For the design of the full library and expression measurement values, see **S3 Table**. Native sequences were chosen to have at least 80% of the polyA site measurements[17] in their first 100bp downstream to the coding region, including the main polyadenylation (highest peak) site. For each gene the 102bp downstream to the coding region was used. A subset of 217 sequences was selected for scanning mutagenesis by uniformly sampling the above set expression distribution[54]. The mutations of the scanning mutagenesis set were done by randomly replacing each nucleotide to one of the other three nucleotides. For all sequences two different 10bp mutations were generated in each position in intervals of 10bp. A small subset of the sequences was also mutated by single 9bp mutations in intervals of 3bp. The set of literature curated elements in non-terminating context was based on Gou et al.[42]. The mutations for modifying the A/T content were done by selecting equal number of modified nucleotides for each target A/T content.

### Discriminative model of positive functional elements in the 3’ end sequences

The mutated subsequences were divided into a positive and a negative set such that the positive set contained subsequences for which a mutation reduces expression to the lower expression peak (expression < 1, **Fig 2D**) and the negative set contained all other subsequences. For each mutated subsequence (instance) its classification was predicted from its mono/di/tri-nucleotide frequencies using a 10-fold cross validation scheme, in which the data was split into 10 subsets and expression was predicted of each subsequence using a model that was trained on the nine subsets that did not include the mutated sequence. Splitting the sequences was done at the level of complete 3’ end sequence, such that subsequences that belong to the same 3’ end were in the same set in each cross validation partition. A logistic regression classifier was learned using the glmnet package from the Tibshirani lab[55], with L1 regularization. The regularization parameter lambda was chosen using internal 10-fold cross validation on the training set.

### Measuring the 3’ end library with induced and un-induced promoter states

To measure the dynamic range of the library in both induced and un-induced promoter states, a yeast culture transformed with the library was grown on SC-URA (synthetic complete media without uracil) 2% Glucose till stationary phase. Stationary culture was then centrifuged, washed, re-suspended and inoculated into two conditions: SC-URA 2% Glucose for the un-induced promoter state and SC-URA 2% Galactose for the induced promoter state. Cells were grown for 4 hours before measurement. A short time was chosen such that the Gal1/10 promoter will display a bi-modal activation distribution[56] and both induced and un-induced states will be measured within the same culture. Flow cytometry was performed with the FACSAria cell sorter (Becton-Dickinson).

### 3' rapid amplification of cDNA ends (3’RACE)

The library was grown in rich media to stationary phase. Stationary cells were then inoculated into fresh synthetic media containing 2% of galactose to induce expression. 10ml of cells in mid-log phase were collected after 6 hours, thoroughly mixed, separated to two replicates, centrifuged and pellet was immediately frozen in liquid nitrogen. RNA was then extracted using Yeast MasterPure kit (Epicenter Biotechnologies) with a long (1 hour) DNAse treatment to avoid contaminations of genomic DNA. YFP specific cDNA was prepared using nested 3′RACE as previously described[57].

### Generating individual 3’ end strains

Chosen native sequences were amplified from the yeast genome with 25bp primer overhang that matched the flanking regions of the SexAI and AvrII restriction sites on the library backbone plasmid. Native sequences were then cloned using Gibson (NEB) cloning to a digested backbone vector (**S16 Fig**). Mutated 3’ end strains were constructed in a similar way using 3 fragments Gibson cloning such that mutated sequences were added in the Gibson overhangs. After the plasmids were generated they were transformed into yeast strain Y8205 and grown in an SC—URA media to select for positive transformed cells.

### Acquisition of bulk time course OD and florescence measurements

Cells were inoculated from stocks of −80°C into SC+2% Glucose-URA and left to grow at 30°C for 48 hours, reaching stationary phase. Next, 5ul were passed into a fresh medium (175ul SC+2% Galactose). Measurements were carried out every ~20 minutes using a robotic system (Tecan Freedom EVO) with a plate reader (Tecan Infinite F500). Each measurement included optical density (filter wavelengths 600 nm, bandwidth 10 nm), YFP fluorescence (excitation 500 nm, emission 540 nm, bandwidths 25/25 nm accordingly) and mCherry fluorescence (excitation 570 nm, emission 630 nm, bandwidths 25/35 nm accordingly). Measurements were done in six replicates

### qPCR analysis for quantification of mRNA levels

Strains were grown in a 96 well plate with 6 replicate wells for each strain in SC+Glu-URA until stationary phase. 5ul of stationary cells were then inoculated into fresh synthetic media—URA (175ul) with 2% galactose till stationary phase and then re-inoculated to induce expression for measurements. Cells were collected after 4.5 hours from mid log phase centrifuged and pellet was immediately frozen in liquid nitrogen. RNA was then extracted using Yeast MasterPure kit (Epicenter Biotechnologies) with a long (1 hour) DNAse treatment to avoid contaminations of genomic DNA. cDNA was prepared using M-MLV reverse transcriptase and random hexamers primers. Quantitative PCR analysis was performed by RT-PCR (StepOnePlus, Applied Biosystems) using ready-mix kit (KAPA, KK4605) with primers spanning the ORF of either YFP (Fw-CCAGAAGGTTATGTTCAA, Rv- CGATTCTATTAACTAAGGTATC) or mCherry (Fw-TGTGGGAGGTGATGTCCAACTTGA, Rv- AGATCAAGCAGAGGCTGAAGCTGA) mRNA molecules in 20 ul volume with triplicate wells for each reaction. Standard curves were prepared by mixing all samples and preparing 4 serial dilutions of 1:5.

## Supporting Information

S1 Fig Identical 3’ end sequences with different barcodes show highly similar expression levels. **(A)** Shown is a boxplot representation of the expression (y-axis) of 87 groups of at least ten identical 3’ end sequences that differ only by their barcodes (x-axis). **(B)** Cumulative distribution of the expression levels relative standard deviation (RSD) of groups of similar 3’ end sequences and different barcodes. The median RSD is 13.2%.
(PDF)

S2 Fig 3’ end sequences mutated by two different 10bp mutation in the same position show highly correlated expression levels. Shown is a comparison of the expression level mediated by 1436 pairs (dots) of 3’ end sequence in which two random modifications of 10bp were introduced in identical positions over the same background sequence. Note that the expression mediated by the two sequences is highly correlated (R^2^ = 0.77, Pearson correlation) suggesting that the dominant effect of the sequence modification is of the removal of the modified sequence and not the addition of the new sequence.
(PDF)

S3 Fig Comparing the effect of mutating 3’ end sequences upstream and downstream to the measured polyadenylation site. A comparison of the expression fold change caused by random mutation of 10bp upstream (orange, 2529 mutation) and downstream (blue, 1067 mutation) to the measured polyadenylation (polyA) site[17]. Mutations upstream to the polyadenylation site cause significantly stronger reduction of the expression level (t-test p<10^–69^).
(PDF)

S4 Fig Estimating the length of the regulatory elements in 3’ end sequences. Shown is the fraction of 172 native 3’ end sequences in which a mutation overlaps a specific nucleotide (meaning, specific position in the 3’ end sequence) reduces expression below 0.8. Each nucleotide was given a score which is the mean expression of all mutated sequence in which the mutation overlaps the its position. The nucleotides are sorted according to their expression from low to high (x-axis). Note that less than 10% of the sequences contain more than 30bp in which a mutation reduces expression below the threshold. This provides an upper bound on the regulatory element length.
(PDF)

S5 Fig Mutations in the main 3’ end functional element reduce expression to a low level independent of wild type expression level. A comparison of the expression levels of 217 native 3’ end sequences (x-axis) to the expression of the sequences with a random 10bp mutation which causes the maximal reduction of expression (y-axis). Each dot is a pair of non-mutated and a mutated native 3’ end sequence. Note that 179 (82.5%) of the mutations reduce expression below 0.8, independent of the non-mutated sequence expression level.
(PDF)

S6 Fig Measurement of YFP expression for individual strains in an arrayed format. YFP expression normalized by RFP over the exponential growth phase for individually cloned wt and mutated sequences. Measurements are shown for a group of short 3’ end sequences chosen from the library **(A)** and longer sequences **(B)**.
(PDF)

S7 Fig Reduction in YFP expression due to mutations of the TA rich element is due is observed also at the level of mRNA. qPCR measurements of YFP mRNA level for the short **(A)** and long **(B)** individually clones sequences. **(C)** and **(D)** shows the correlation between the mRNA and protein levels for short and long sequences respectively.
(PDF)

S8 Fig Mean nucleotide composition and mean effect of mutation relative to the polyadenylation site. **(A)** Shown is the mean TA,T,A di/mono-nucleotide composition in 20bp sliding windows. All 3’ end sequences are aligned by the strongest polyadenylation site[17]. Estimated positions of transcription termination elements previously described in the literature[19] are marked by gray lines. **(B)** Shown is the mean effect of a mutation in each position using similar sequence alignment and sliding windows as **(A)**. While TA composition which corresponds to the efficiency element co-occurs with a large reduction in expression due to mutations, A and T composition which corresponds to the positioning and cleavage sites do not.
(PDF)

S9 Fig A comparison of measured native 3’ end sequences mediated expression values to endogenous gene expression measurements. Correlation between our measurements of expression levels mediated by native 3’ end sequences (x-axis) to **(A)** mRNA abundance[37], **(B)** protein abundance[39] and **(C)** mRNA half life[40] of the corresponding endogenous genes.
(PDF)

S10 Fig Mutations in efficiency element (EE) and position element (PE) result in a significant reduction in expression. Expression distribution of 3’ end sequences mutated in EE, PE and cleavage site[58] compared to the expression distribution of 21 non-mutated sequences (identical except with different 11bp barcodes). Both mutations in EE and PE reduce expression significantly. However, while most mutation in EE have a very strong effect on expression, mutations in PE show much minor effect.
(PDF)

S11 Fig Mutations of known transcription termination elements in native 3’ end sequences. Shows the expression (gray bars) of 3’ end sequences in which single/double bp mutations were introduced in putative termination motifs that were identified computationally[44] (EE top panels, PE bottom panel). Each single bp mutation was introduced in a separate sequence. The thick black vertical line and boxplot represent the median and distribution of identical 21 non-mutated 3’ end sequences with different barcodes.
(PDF)

S12 Fig Mutating known transcription termination elements in native 3’ end sequences. Expression distribution of 3’ end sequences mutated in efficiency element (EE) and positioning element (PE) in native sequences in which these elements where computationally identified compared to the expression distribution of identical 21 non-mutated sequences except with different barcodes. P values of t-test comparison of each mutated sequences group to the non-mutated are presented in the legend. Sequences are equally divided into bins based on expression such that each bin contains all sequences within a range of 0.6. Three out of four genes show a significant reduction in expression when mutating the EE (p<0.05).
(PDF)

S13 Fig Correlations between the A/T content of different regions of the context sequence surrounding known mRNA 3’ end processing motifs and expression. Shown is the correlation between expression and A/T content of different region of the sequences surrounding transcription elements described in the literature. Results are shown for four different native sequences in which we identify the efficiency element (EE), position element (PE) and cleavage site as described at Tian et al.[44] and a de-novo designed sequence (Synthetic context). In each sequence region of each tested 3’ end sequence we generated a total of XX mutations that sampled the A/T% space uniformly. Each point shows the Pearson correlation (R^2^, y-axis) between A/T content of these mutated sequences in a specific 3’ end region (x-axis) and their expression (y-axis) across the different 3’ end sequences (marker symbol).
(PDF)

S14 Fig Co-occurrence of the mutation that cases the maximal reduction of expression with Nrd1 and Nab3 binding sites. **(A)** Heat map showing the mean effect of a mutation as a function of location in the 3’ end sequence, the mean measured polyadenylation site[17] (black lozenge) and Nrd1 sites[45] (purple star). Each row represents one sequence and the color represents the mean expression fold change across two replicates between the mutated to wild type sequences. Rows are sorted by the location of the maximal affecting mutation **(B)** similar to **(A)** except Nab3 sites[45] (purple star). **(C)** A cumulative distribution of the minimal distance between the middle of the mutation which causes the maximal reduction of expression level and the Nrd1/Nab3 binding site across the tested native 3’ end sequences. For comparison, the average cumulative distribution of 10,000 random permutations of Nrd1/Nab3 sites is also illustrated (dotted lines). The average minimal distance of Nrd1 site from the mutation is smaller than in all permutations (p<10^–4^) and is smaller than 8bp in 36% of the 3’ end sequences. This suggests a possible link between Nrd1 and the pre-mRNA 3’ end processing motif.
(PDF)

S15 Fig Measuring the expression mediated by 68 RNA binding protein motifs curated from the literature in context with predicted open/close RNA 2D structure. **(A)** The expression of 3’ end sequences containing one of 68 RNA binding protein (RBP) motifs curated from the literature[46] (x-axis) in contexts with predicts open (green) or close (red) RNA 2D structures[59]. All 3’ end sequences contained a transcription termination sequence at their 3’ end. **(B)** Shown are the t-test p values for the open structure contexts having higher expression than the close structures. **(C)** The average expression of contexts containing an RBP motif with the average number of dTdA that its placement in the context add to the 3’ end sequence. Notice that no RBP motif showed higher expression in open contexts, however the average expression mediated by 3’ end sequences that contain it is highly correlated with its dTdA content.
(PDF)

S16 Fig Plasmid sequence. Illustration of the plasmid used as a backbone for cloning the library.
(PNG)

S1 Table Context sequences. Backbone 3’ end sequences used for creation of synthetic and mutated sequences.
(XLSX)

S2 Table Element sequences. Short element sequences used for creation of synthetic and mutated sequences.
(XLSX)

S3 Table Library sequences. Full list of all library sequences with detailed description for each sequence and measured expression values.
(XLSX)

S4 Table Individually clones sequences. Sequences used for validation in arrayed format measurements.
(XLSX)
