# Supplementary figures and images for: Systematic Dissection of the Sequence Determinants of Gene 3’ End Mediated Expression Control
Source: PLoS Genet. 2015 Apr 15;11(4):e1005147. doi: 10.1371/journal.pgen.1005147 (PMC4398552; doi:10.1371/journal.pgen.1005147)

A

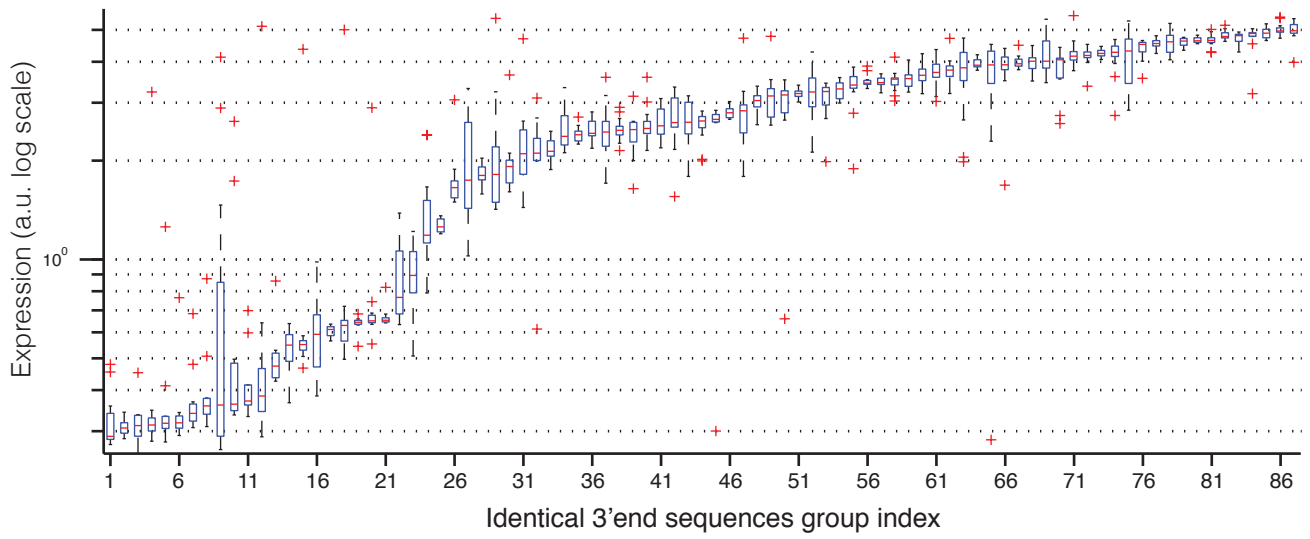

B

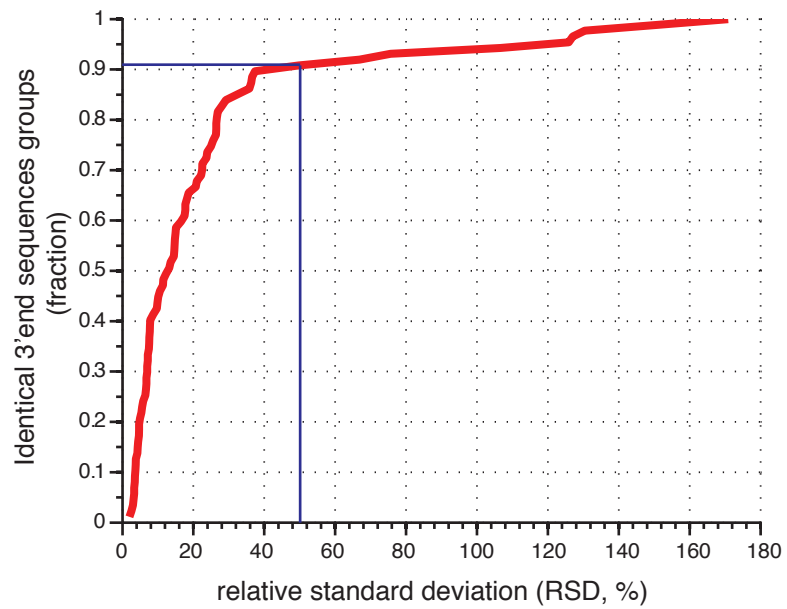

Supplement: S1 Fig — (A) Shown is a boxplot representation of the expression (y-axis) of 87 groups of at least ten identical 3’ end sequences that differ only by their barcodes (x-axis). (B) Cumulative distribution of the expression levels relative standard deviation (RSD) of groups of similar 3’ end sequences and different barcodes. The median RSD is 13.2%. (PDF) [file pgen.1005147.s001.pdf]

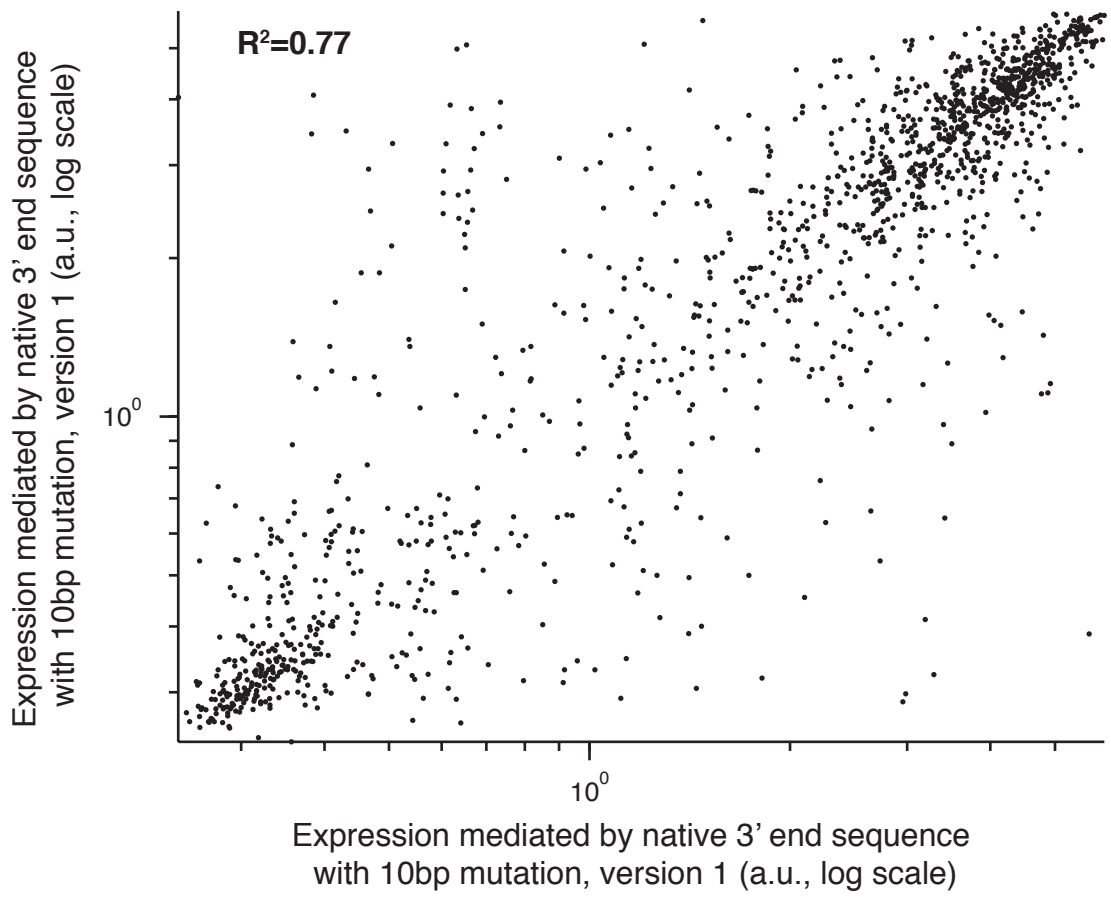

Supplement: S2 Fig — Shown is a comparison of the expression level mediated by 1436 pairs (dots) of 3’ end sequence in which two random modifications of 10bp were introduced in identical positions over the same background sequence. Note that the expression mediated by the two sequences is highly correlated (R2 = 0.77, Pearson correlation) suggesting that the dominant effect of the sequence modification is of the removal of the modified sequence and not the addition of the new sequence. (PDF) [file pgen.1005147.s002.pdf]

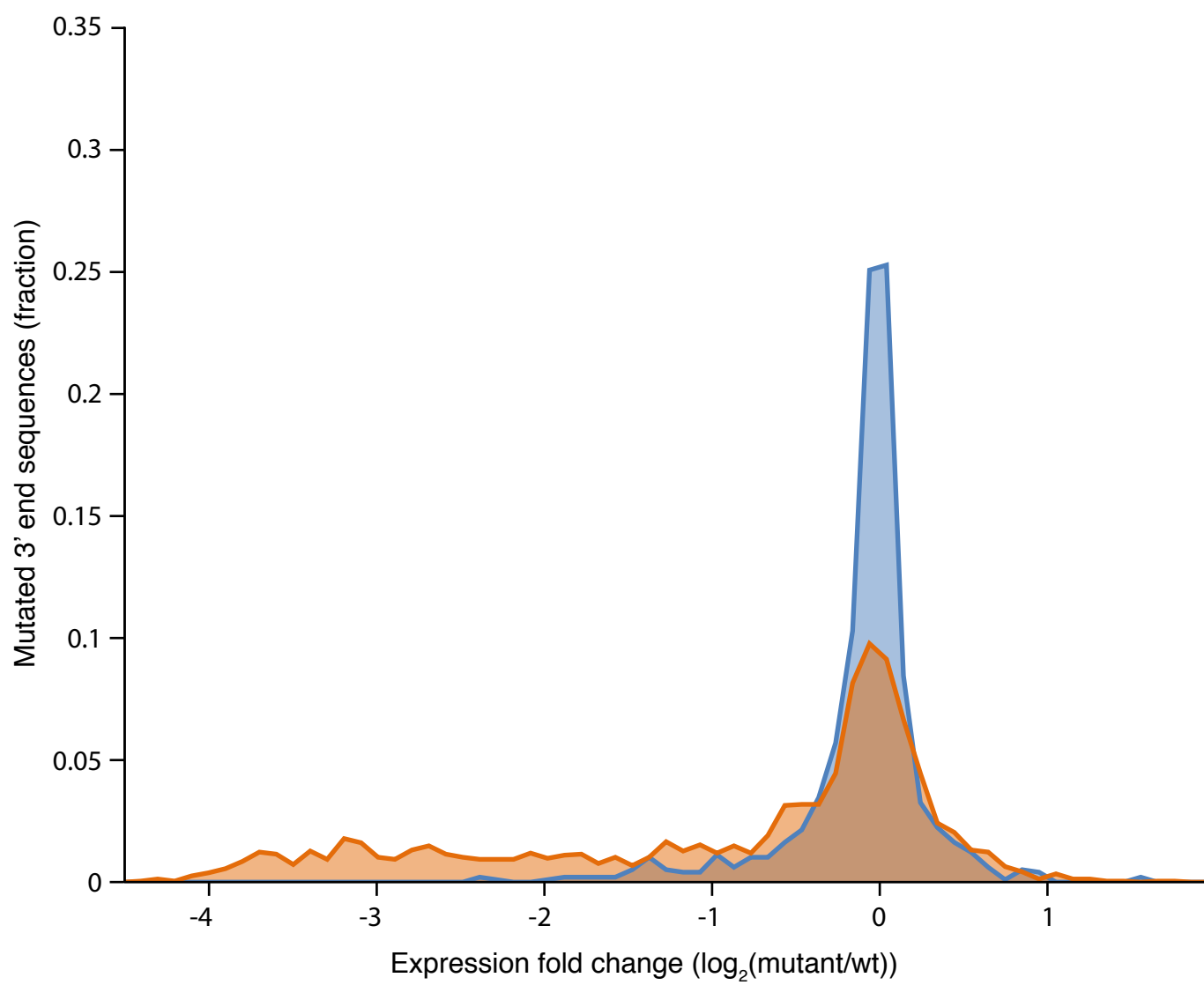

Supplement: S3 Fig — A comparison of the expression fold change caused by random mutation of 10bp upstream (orange, 2529 mutation) and downstream (blue, 1067 mutation) to the measured polyadenylation (polyA) site[17]. Mutations upstream to the polyadenylation site cause significantly stronger reduction of the expression level (t-test p<10–69). (PDF) [file pgen.1005147.s003.pdf]

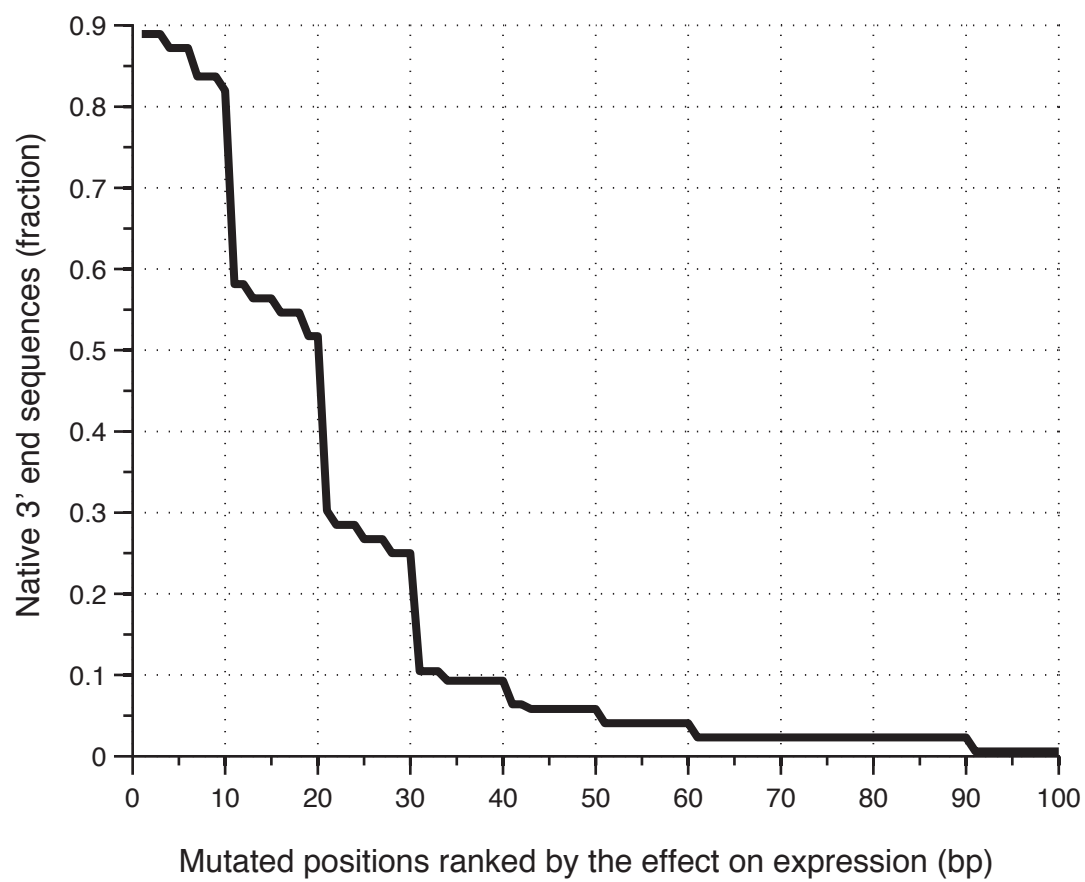

Supplement: S4 Fig — Shown is the fraction of 172 native 3’ end sequences in which a mutation overlaps a specific nucleotide (meaning, specific position in the 3’ end sequence) reduces expression below 0.8. Each nucleotide was given a score which is the mean expression of all mutated sequence in which the mutation overlaps the its position. The nucleotides are sorted according to their expression from low to high (x-axis). Note that less than 10% of the sequences contain more than 30bp in which a mutation reduces expression below the threshold. This provides an upper bound on the regulatory element length. (PDF) [file pgen.1005147.s004.pdf]

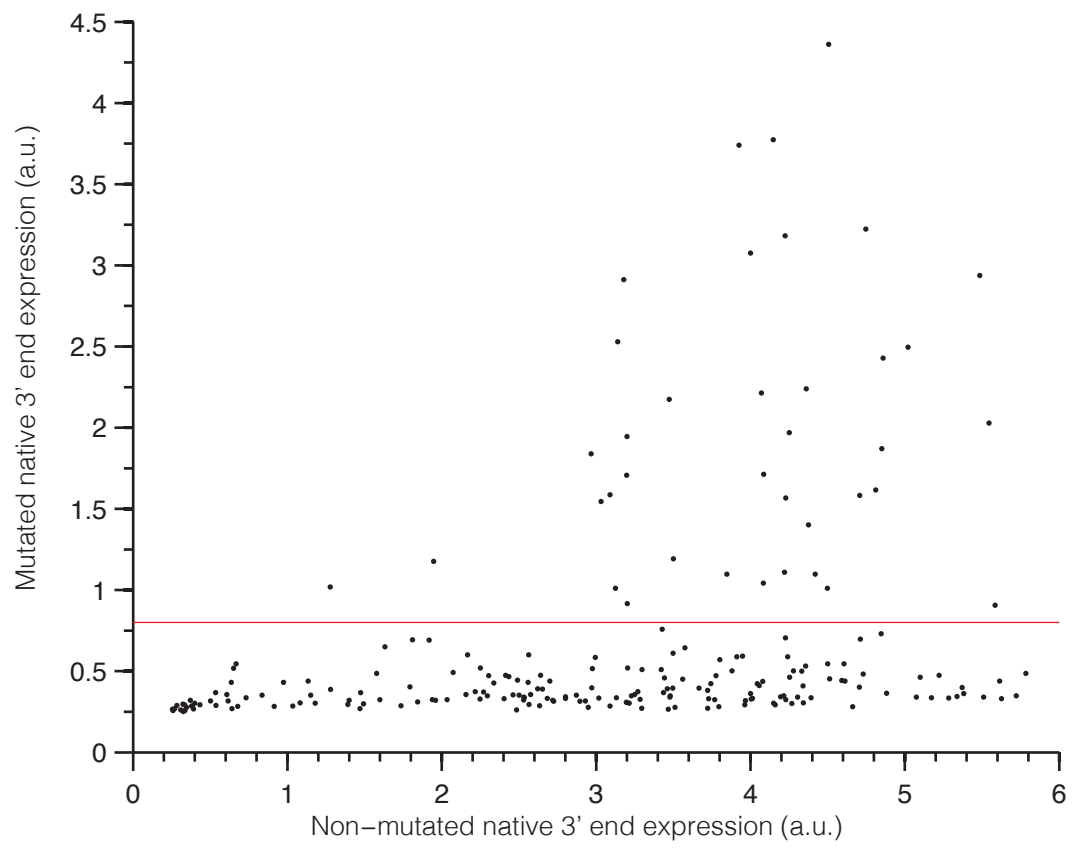

Supplement: S5 Fig — A comparison of the expression levels of 217 native 3’ end sequences (x-axis) to the expression of the sequences with a random 10bp mutation which causes the maximal reduction of expression (y-axis). Each dot is a pair of non-mutated and a mutated native 3’ end sequence. Note that 179 (82.5%) of the mutations reduce expression below 0.8, independent of the non-mutated sequence expression level. (PDF) [file pgen.1005147.s005.pdf]

**A**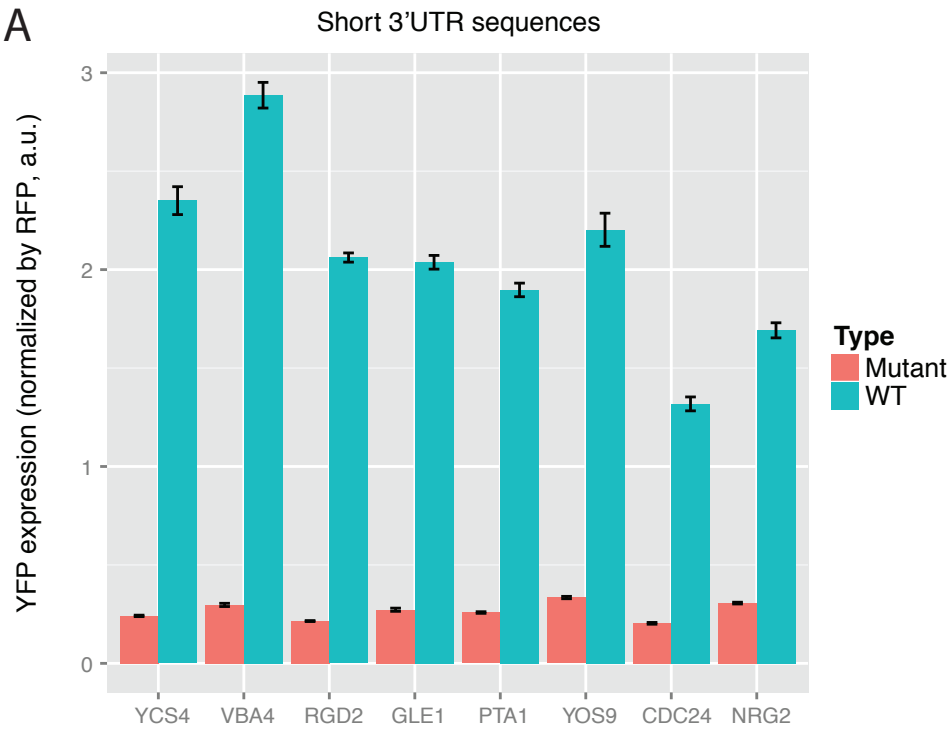**B**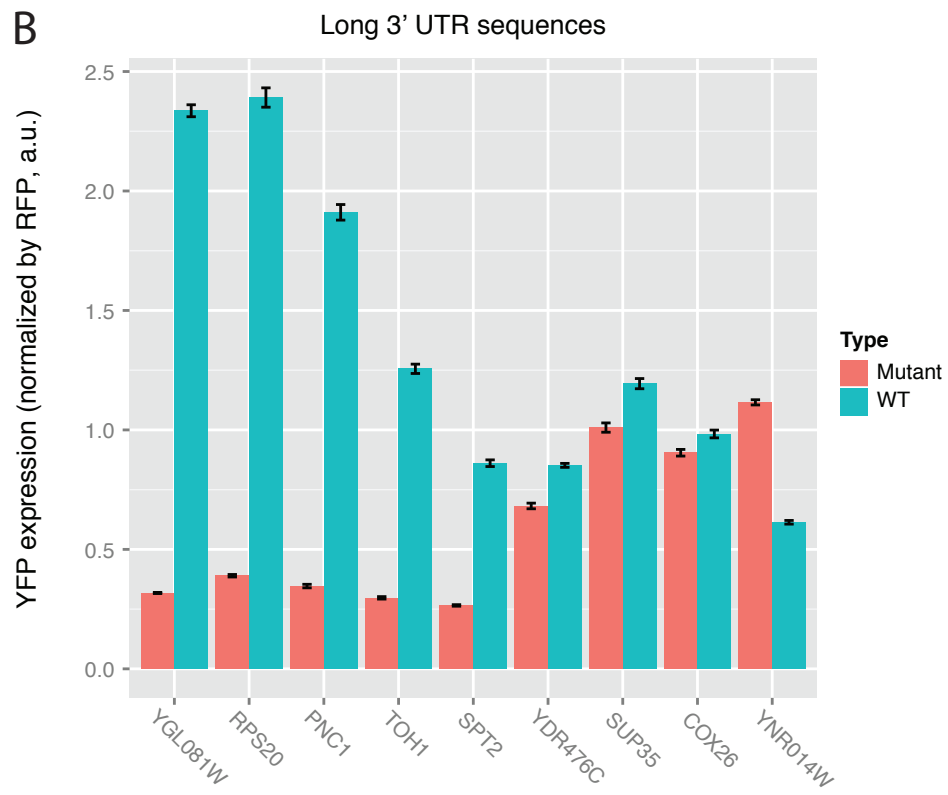

Supplement: S6 Fig — YFP expression normalized by RFP over the exponential growth phase for individually cloned wt and mutated sequences. Measurements are shown for a group of short 3’ end sequences chosen from the library (A) and longer sequences (B). (PDF) [file pgen.1005147.s006.pdf]

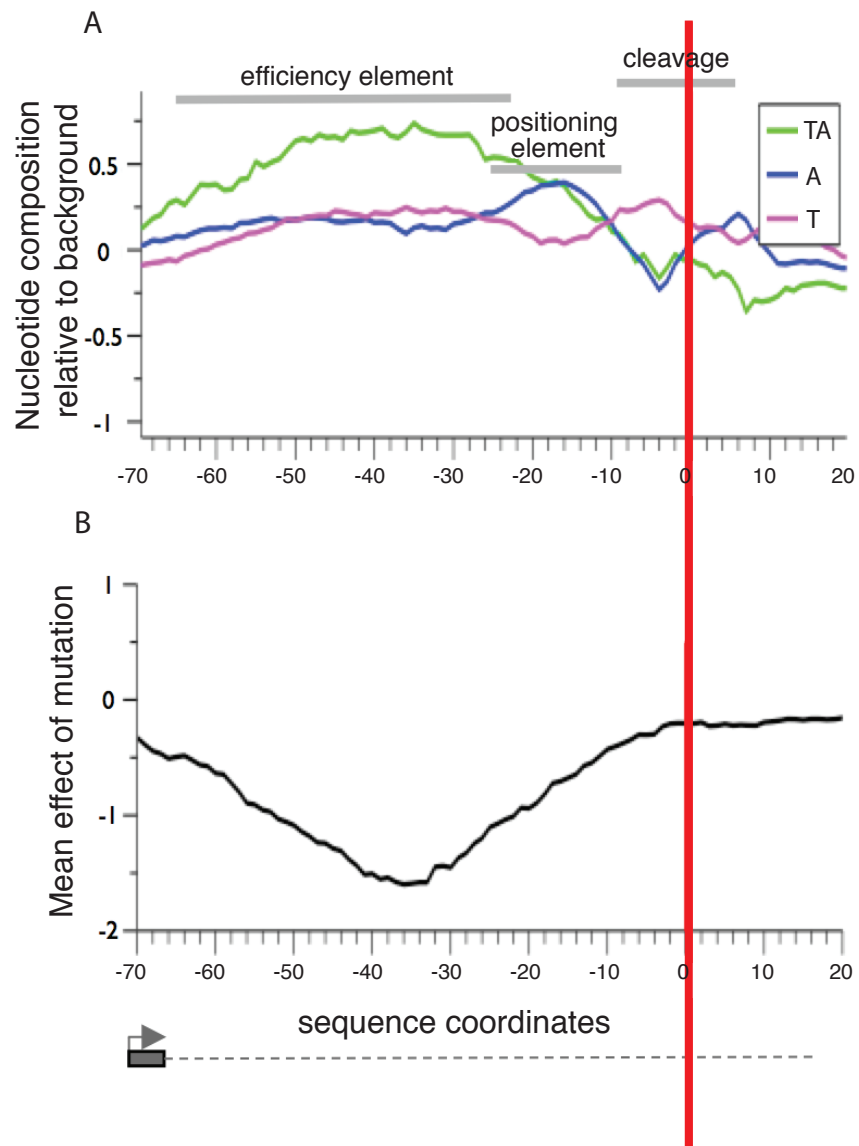

Supplement: S8 Fig — (A) Shown is the mean TA,T,A di/mono-nucleotide composition in 20bp sliding windows. All 3’ end sequences are aligned by the strongest polyadenylation site[17]. Estimated positions of transcription termination elements previously described in the literature[19] are marked by gray lines. (B) Shown is the mean effect of a mutation in each position using similar sequence alignment and sliding windows as (A). While TA composition which corresponds to the efficiency element co-occurs with a large reduction in expression due to mutations, A and T composition which corresponds to the positioning and cleavage sites do not. (PDF) [file pgen.1005147.s008.pdf]

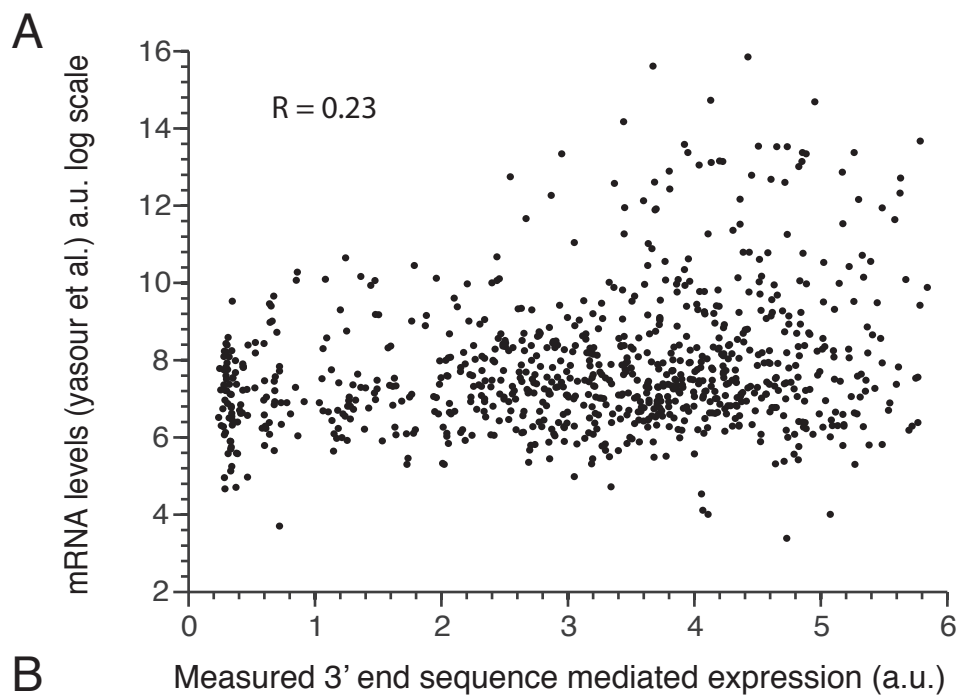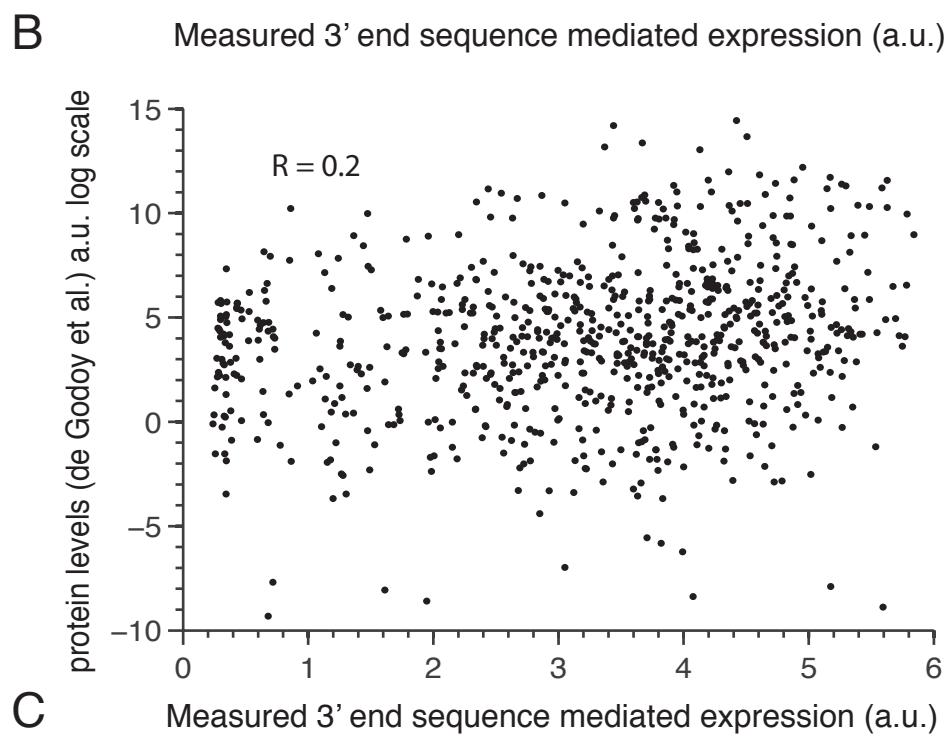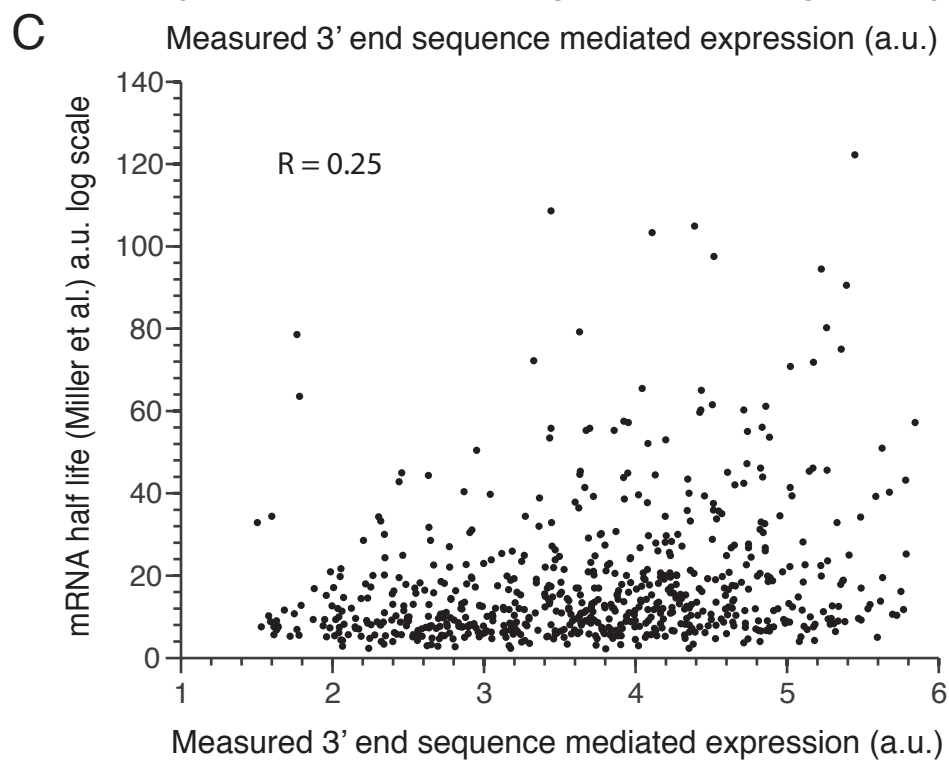

Supplement: S9 Fig — Correlation between our measurements of expression levels mediated by native 3’ end sequences (x-axis) to (A) mRNA abundance[37], (B) protein abundance[39] and (C) mRNA half life[40] of the corresponding endogenous genes. (PDF) [file pgen.1005147.s009.pdf]

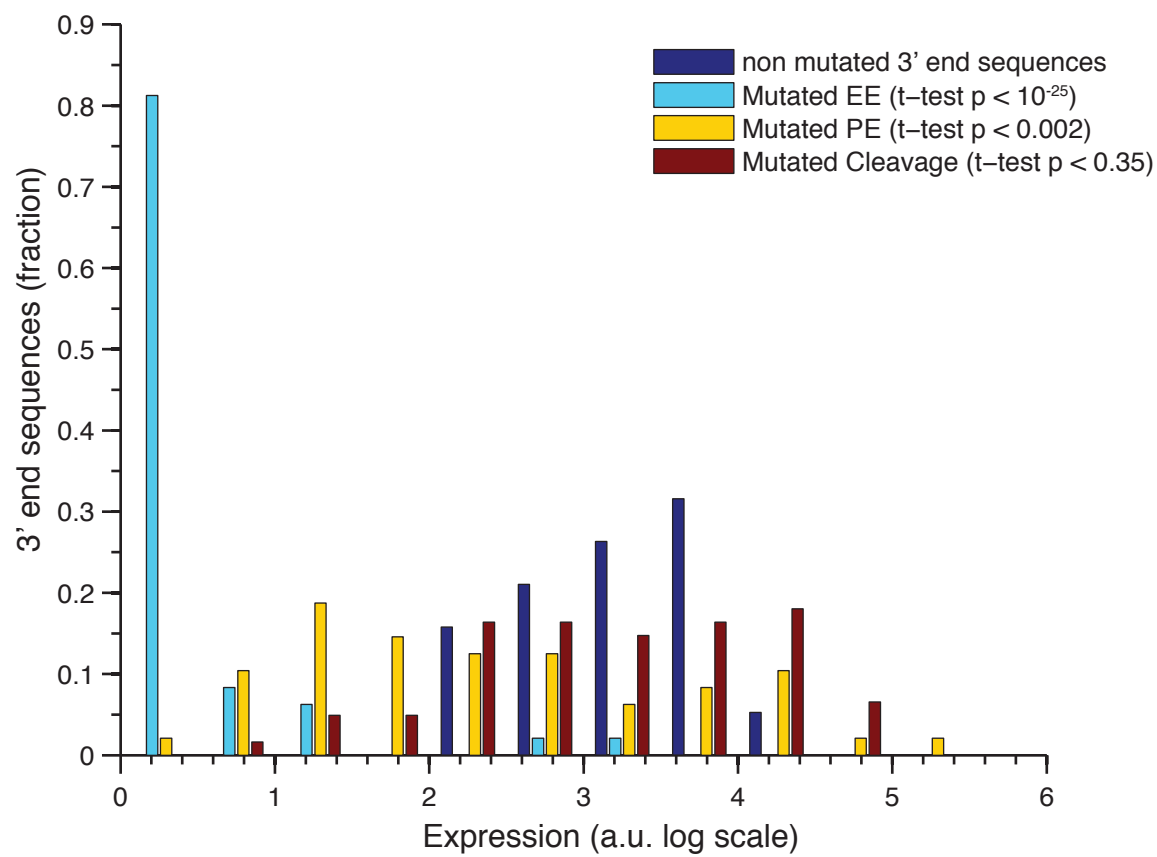

Supplement: S10 Fig — Expression distribution of 3’ end sequences mutated in EE, PE and cleavage site[58] compared to the expression distribution of 21 non-mutated sequences (identical except with different 11bp barcodes). Both mutations in EE and PE reduce expression significantly. However, while most mutation in EE have a very strong effect on expression, mutations in PE show much minor effect. (PDF) [file pgen.1005147.s010.pdf]

YDR286C

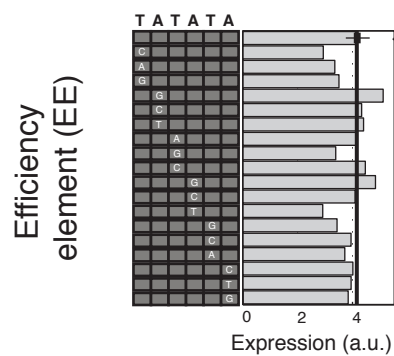COX18  
(YGR062C)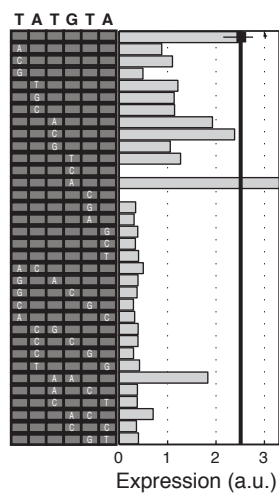BCK1  
(YJL095W)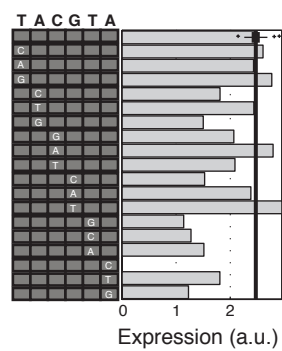PDC1  
(YLR044C)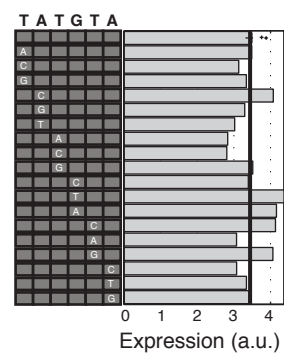Positioning  
element (PE)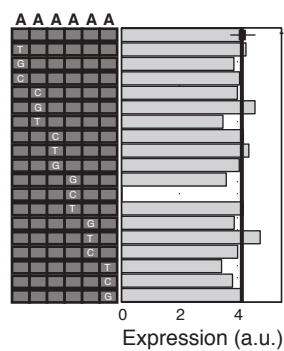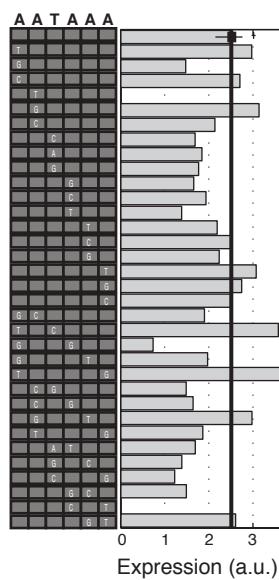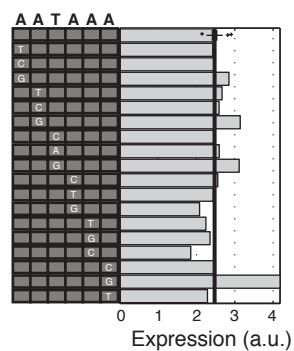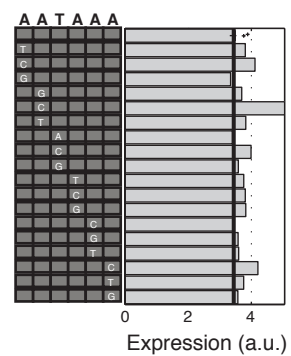

Supplement: S11 Fig — Shows the expression (gray bars) of 3’ end sequences in which single/double bp mutations were introduced in putative termination motifs that were identified computationally[44] (EE top panels, PE bottom panel). Each single bp mutation was introduced in a separate sequence. The thick black vertical line and boxplot represent the median and distribution of identical 21 non-mutated 3’ end sequences with different barcodes. (PDF) [file pgen.1005147.s011.pdf]

YDR286C

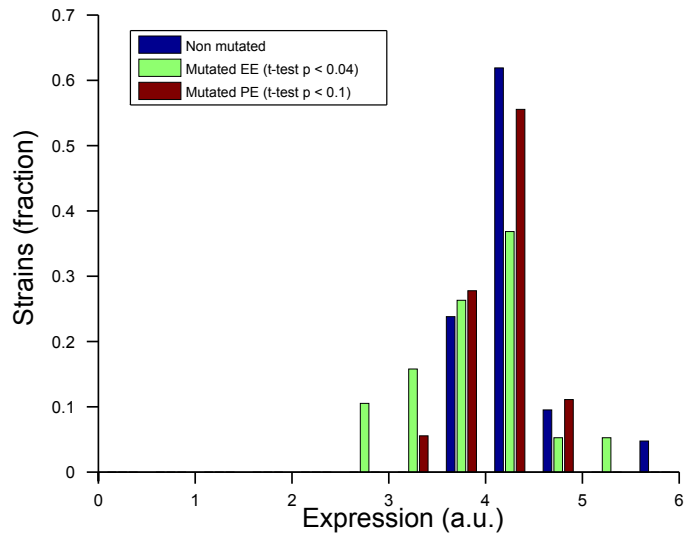

YLR044C

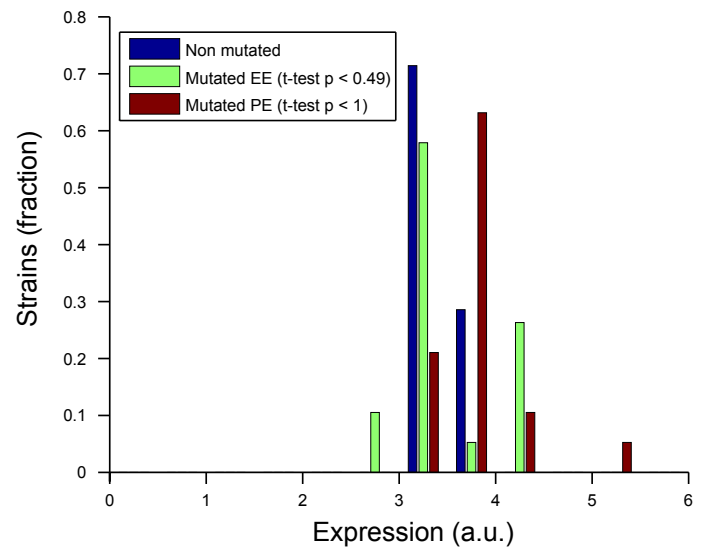

YJL095W

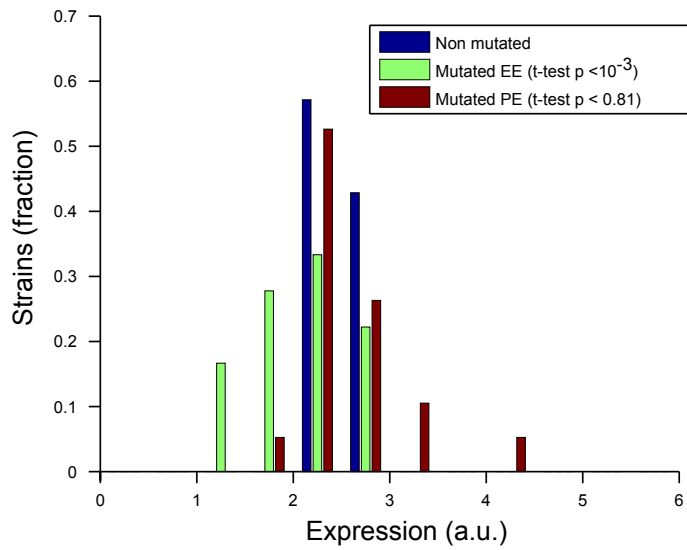

YGR062C

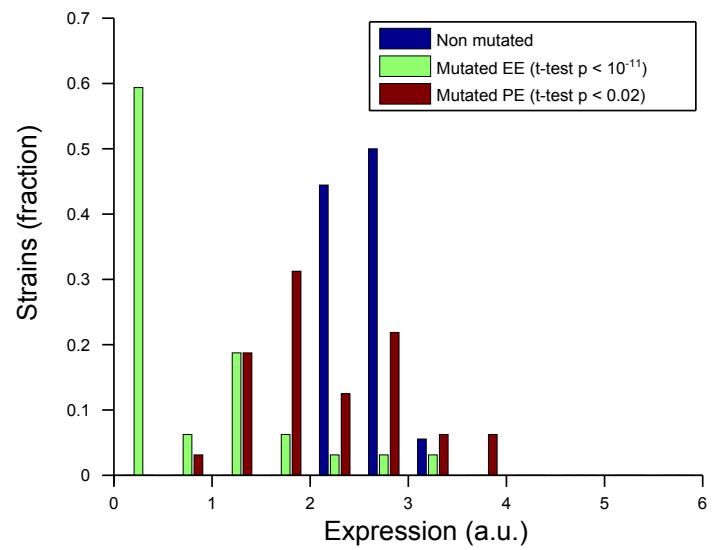

Supplement: S12 Fig — Expression distribution of 3’ end sequences mutated in efficiency element (EE) and positioning element (PE) in native sequences in which these elements where computationally identified compared to the expression distribution of identical 21 non-mutated sequences except with different barcodes. P values of t-test comparison of each mutated sequences group to the non-mutated are presented in the legend. Sequences are equally divided into bins based on expression such that each bin contains all sequences within a range of 0.6. Three out of four genes show a significant reduction in expression when mutating the EE (p<0.05). (PDF) [file pgen.1005147.s012.pdf]

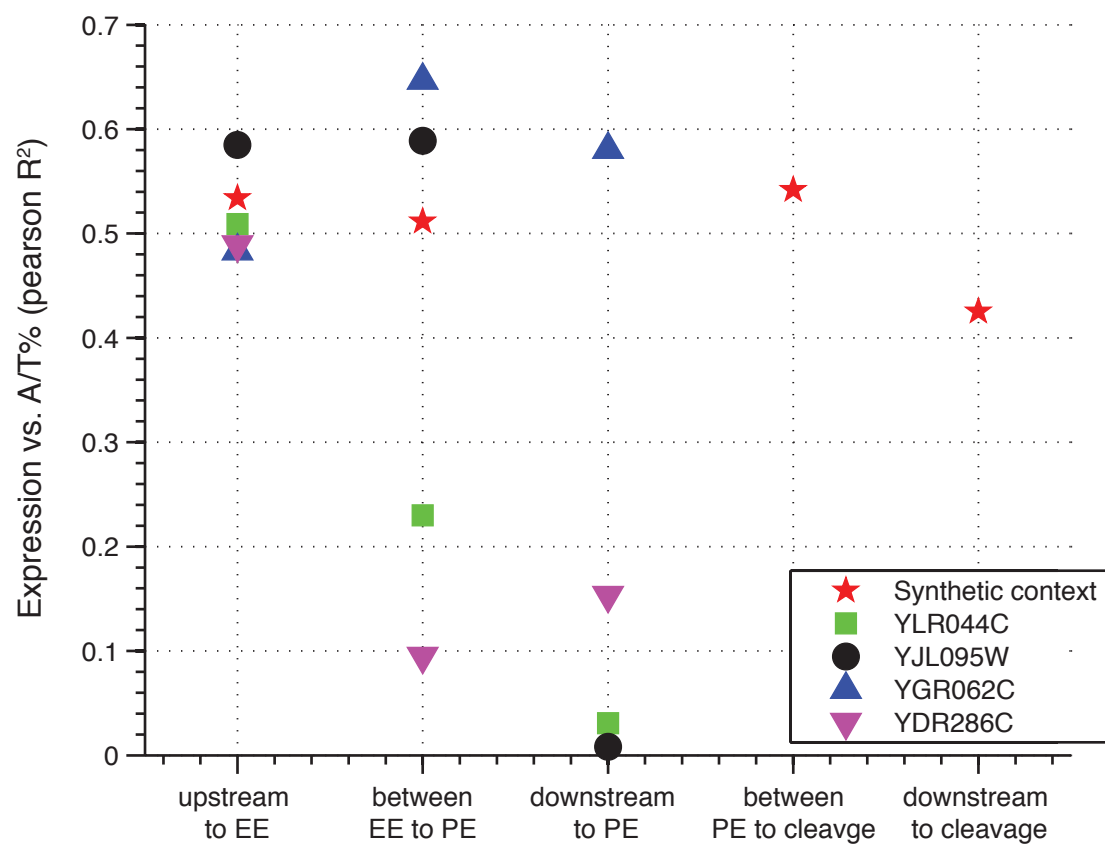

Supplement: S13 Fig — Shown is the correlation between expression and A/T content of different region of the sequences surrounding transcription elements described in the literature. Results are shown for four different native sequences in which we identify the efficiency element (EE), position element (PE) and cleavage site as described at Tian et al.[44] and a de-novo designed sequence (Synthetic context). In each sequence region of each tested 3’ end sequence we generated a total of XX mutations that sampled the A/T% space uniformly. Each point shows the Pearson correlation (R2, y-axis) between A/T content of these mutated sequences in a specific 3’ end region (x-axis) and their expression (y-axis) across the different 3’ end sequences (marker symbol). (PDF) [file pgen.1005147.s013.pdf]

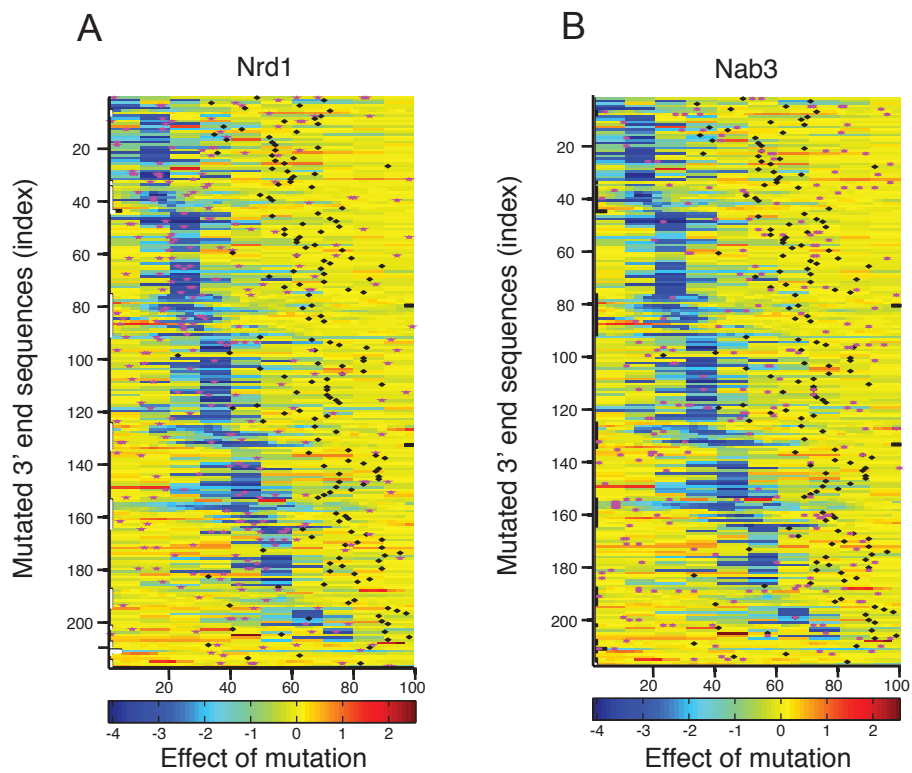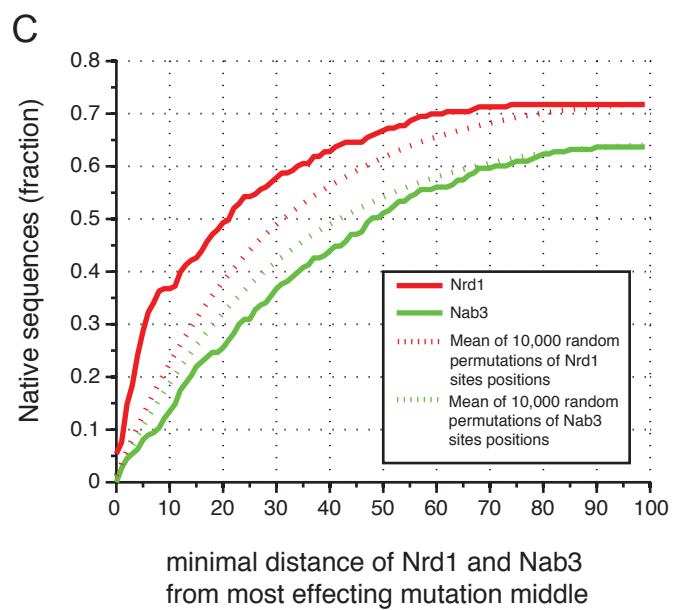

Supplement: S14 Fig — (A) Heat map showing the mean effect of a mutation as a function of location in the 3’ end sequence, the mean measured polyadenylation site[17] (black lozenge) and Nrd1 sites[45] (purple star). Each row represents one sequence and the color represents the mean expression fold change across two replicates between the mutated to wild type sequences. Rows are sorted by the location of the maximal affecting mutation (B) similar to (A) except Nab3 sites[45] (purple star). (C) A cumulative distribution of the minimal distance between the middle of the mutation which causes the maximal reduction of expression level and the Nrd1/Nab3 binding site across the tested native 3’ end sequences. For comparison, the average cumulative distribution of 10,000 random permutations of Nrd1/Nab3 sites is also illustrated (dotted lines). The average minimal distance of Nrd1 site from the mutation is smaller than in all permutations (p<10–4) and is smaller than 8bp in 36% of the 3’ end sequences. This suggests a possible link between Nrd1 and the pre-mRNA 3’ end processing motif. (PDF) [file pgen.1005147.s014.pdf]

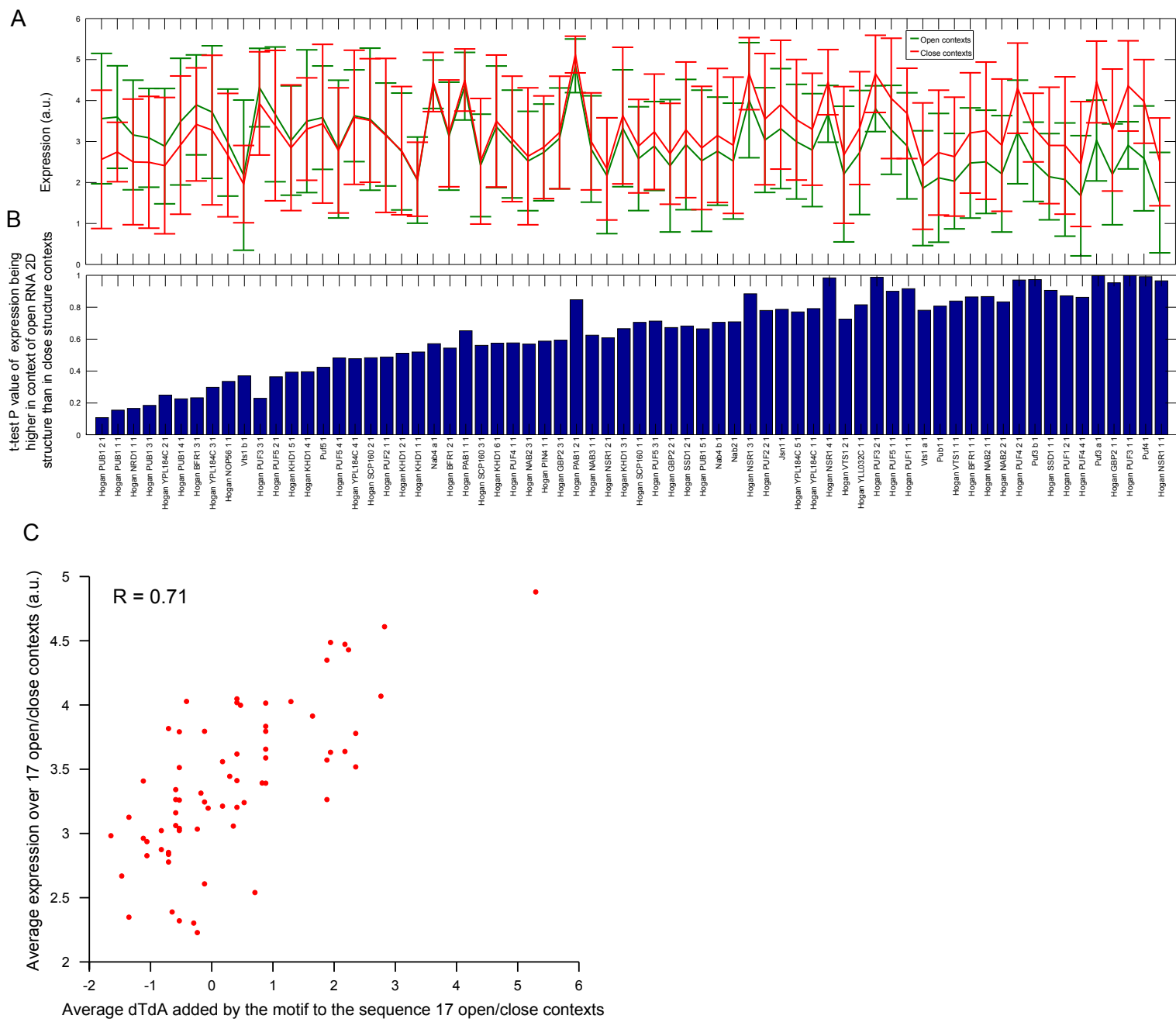

Supplement: S15 Fig — (A) The expression of 3’ end sequences containing one of 68 RNA binding protein (RBP) motifs curated from the literature[46] (x-axis) in contexts with predicts open (green) or close (red) RNA 2D structures[59]. All 3’ end sequences contained a transcription termination sequence at their 3’ end. (B) Shown are the t-test p values for the open structure contexts having higher expression than the close structures. (C) The average expression of contexts containing an RBP motif with the average number of dTdA that its placement in the context add to the 3’ end sequence. Notice that no RBP motif showed higher expression in open contexts, however the average expression mediated by 3’ end sequences that contain it is highly correlated with its dTdA content. (PDF) [file pgen.1005147.s015.pdf]

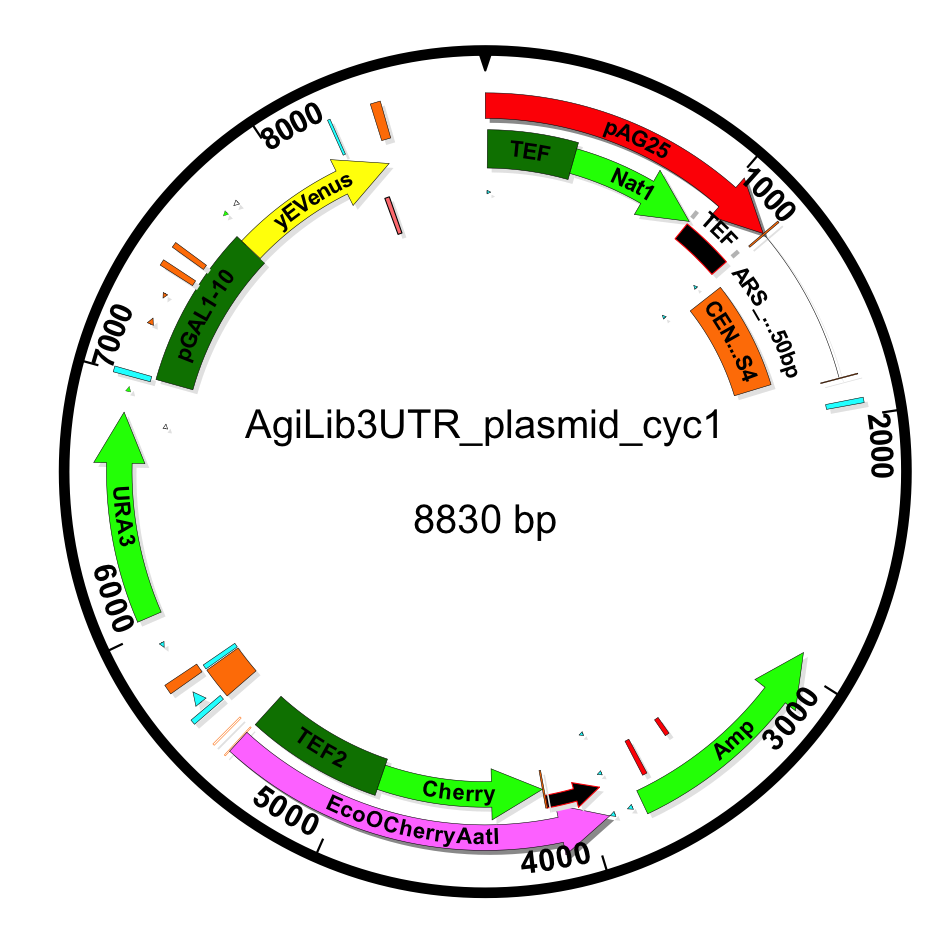

Supplement: S16 Fig — Illustration of the plasmid used as a backbone for cloning the library. (PNG) [file pgen.1005147.s016.png]
