# Supplementary material for: Systematic Dissection of the Sequence Determinants of Gene 3’ End Mediated Expression Control
Source: PLoS Genet. 2015 Apr 15;11(4):e1005147. doi: 10.1371/journal.pgen.1005147 (PMC4398552; doi:10.1371/journal.pgen.1005147)
Supplement: S7 Fig — qPCR measurements of YFP mRNA level for the short (A) and long (B) individually clones sequences. (C) and (D) shows the correlation between the mRNA and protein levels for short and long sequences respectively. (PDF) [file pgen.1005147.s007.pdf]

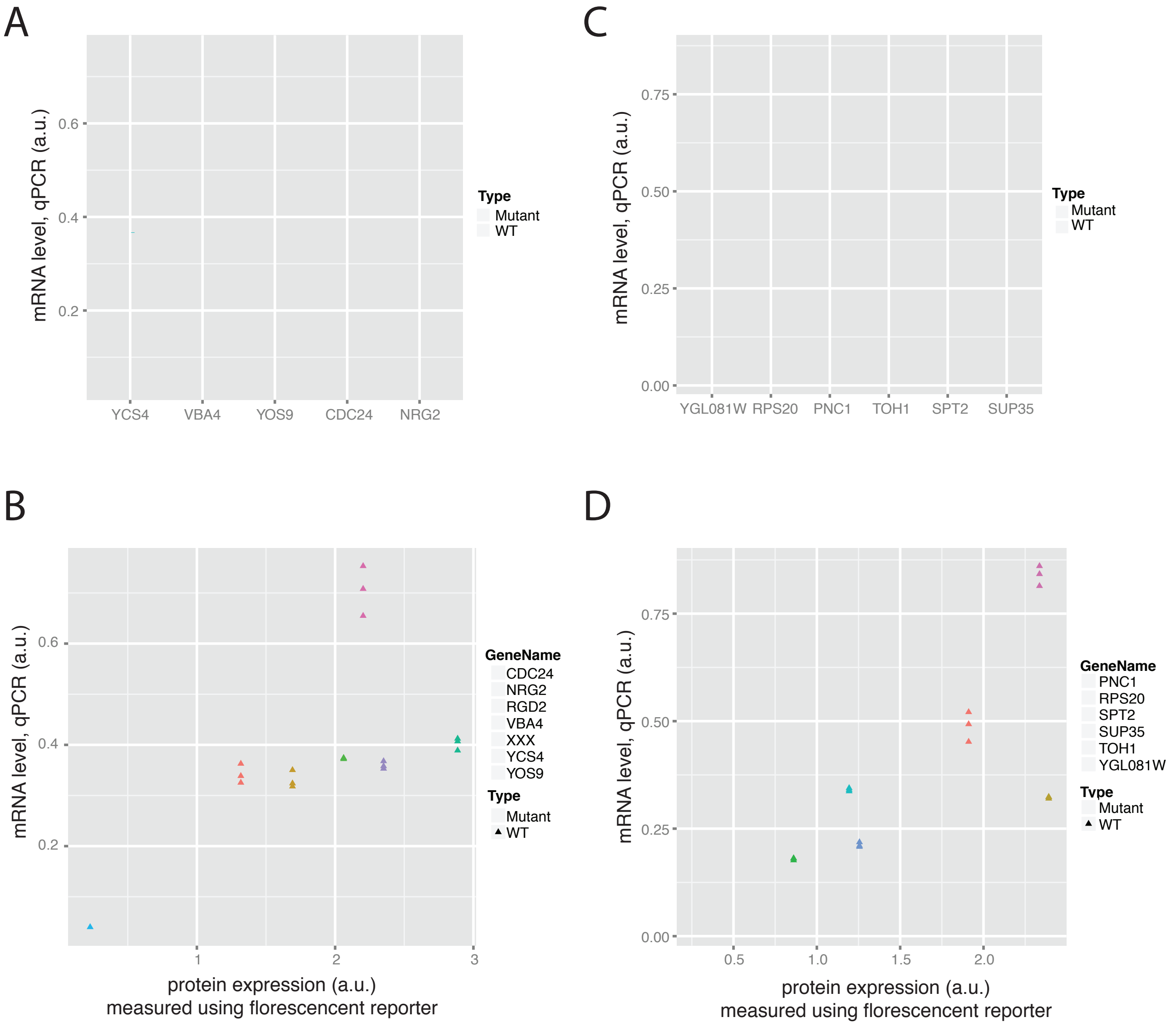
A
mRNA level, qPCR (a.u.)
0.6
0.4
0.2
YCS4
VBA4
YOS9
CDC24
NRG2
Type
Mutant
WT
C
mRNA level, qPCR (a.u.)
0.75
0.50
0.25
0.00
YGL081W
RPS20
PNC1
TOH1
SPT2
SUP35
Type
Mutant
WT
B
mRNA level, qPCR (a.u.)
0.6
0.4
0.2
1
2
3
protein expression (a.u.)
measured using florescencent reporter
GeneName
CDC24
NRG2
RGD2
VBA4
XXX
YCS4
YOS9
Type
Mutant
WT
D
mRNA level, qPCR (a.u.)
0.75
0.50
0.25
0.00
0.5
1.0
1.5
2.0
protein expression (a.u.)
measured using florescencent reporter
GeneName
PNC1
RPS20
SPT2
SUP35
TOH1
YGL081W
Type
Mutant
WT
